# Development and Validation of a Prognostic Index Based on Genes Participating in Autophagy in Patients With Lung Adenocarcinoma

**DOI:** 10.3389/fonc.2021.799759

**Published:** 2022-01-25

**Authors:** Zi-Xuan Wu, Xuyan Huang, Min-Jie Cai, Pei-Dong Huang, Zunhui Guan

**Affiliations:** ^1^Guangzhou University of Chinese Medicine, Guangzhou, China; ^2^Shantou Health School, Shantou, China; ^3^Yunnan University of Chinese Medicine, Kunming, China; ^4^Kunming Municipal Hospital of Traditional Chinese Medicine, Kunming, China

**Keywords:** lung adenocarcinoma (LUAD), TCGA and GEO dataset, immunity, m^6^a, and immune checkpoint, bioinformatics analysis, genes participating in autophagy

## Abstract

**Background:**

Lung adenocarcinoma (LUAD) is a deadly respiratory system malignancy with poor prognosis. Autophagy is essential for the beginning, development, and therapy resistance of cancer. However, the expression of genes participating in autophagy in LUAD and their associations with prognosis remain unclear.

**Methods:**

Predictive genes participating in autophagy in LUAD samples from The Cancer Genome Atlas (TCGA) and Gene Expression Omnibus (GEO) datasets were investigated. TCGA and GEO cohorts were divided into two risk groups, while the low-risk group having a longer overall survival (OS) time. This article aims to point out the interaction between genes participating in autophagy and immune function, immune checkpoints, and m^6^a in LUAD. The prediction model was designed for exploring least absolute shrinkage and selection operator (LASSO) regression. It has been revealed that gene expression and autophagy are inextricably connected.

**Results:**

Genes participating in autophagy were shown to be somewhat overexpressed in the high-risk group even though no different clinical symptoms were present, indicating that they might be used in a model to predict LUAD prognosis. The majority of genes participating in autophagy prognostic signatures controlled immunological and tumor-related pathways, according to gene set enrichment analysis (GSEA). *KRT6A*, *KYNU*, *IGFBP1*, *DKK1*, *PKP2*, *PLEK2*, *GAPDH*, *FLNC*, and *NTSR1* might be related to the oncology process for LUAD patients. *CERS4*, *CMAHP*, and *PLEKHB1* have been identified as being associated with low risk in patients with LUAD. Furthermore, the immune function and m^6^a gene expression differed significantly between the two groups.

**Conclusions:**

Genes participating in autophagy are connected to the development and progression of LUAD. LUAD patients’ prognoses are often foreseen utilizing matched prognostic models. Genes participating in autophagy in LUAD may be therapeutic targets that ought to be investigated more.

## Introduction

Lung adenocarcinoma (LUAD) may be a leading reason for cancer-related death globally. LUAD is classified into two categories in microscopic anatomy, which differ clinically (1, 2). Significantly, more than half of LUAD patients had metastases at the time of diagnosis. Despite this, the absence of precise biomarkers for early tumor identification, likewise as restricted preclinical models, has obstructed prospering LUAD treatment (3, 4). To avoid the onset and development of LUAD, more molecular identification is essential for its fundamental and clinical research, likewise as the identification of novel and effective LUAD prognostic indicators.

Autophagy can be a cell-renewal mechanism that depends on the breakdown of cytoplasmic proteins or lysosome organelles. As a sort of death, it has received loads of attention and dialogue in recent years (5, 6). Yoshinori Ohsumi (7) highlighted the essential principle: autophagy is essential for eliminating “garbage” from cells, preventing aberrant death and protecting traditional cell functioning. It is a cell self-defense and self-renewal method that depends on lysosomes to destroy their organelles or proteins (8, 9). A growing variety of studies have discovered that autophagy is crucial in maintaining the intracellular environment’s integrity and participates in cellular processes (10). In distinction, alternative investigations have discovered that many diseases, including cancer and respiratory organ malady, are joined to enhance or reduce the levels of autophagy (11). Despite contradictory evidence showing that autophagy is thought to promote oncogenesis and cancer spread. Rare sequence-based studies on aberrant gene expression and its relationship to overall survival (OS) in LUAD patients with autophagy were conducted.

**Figure 1:**
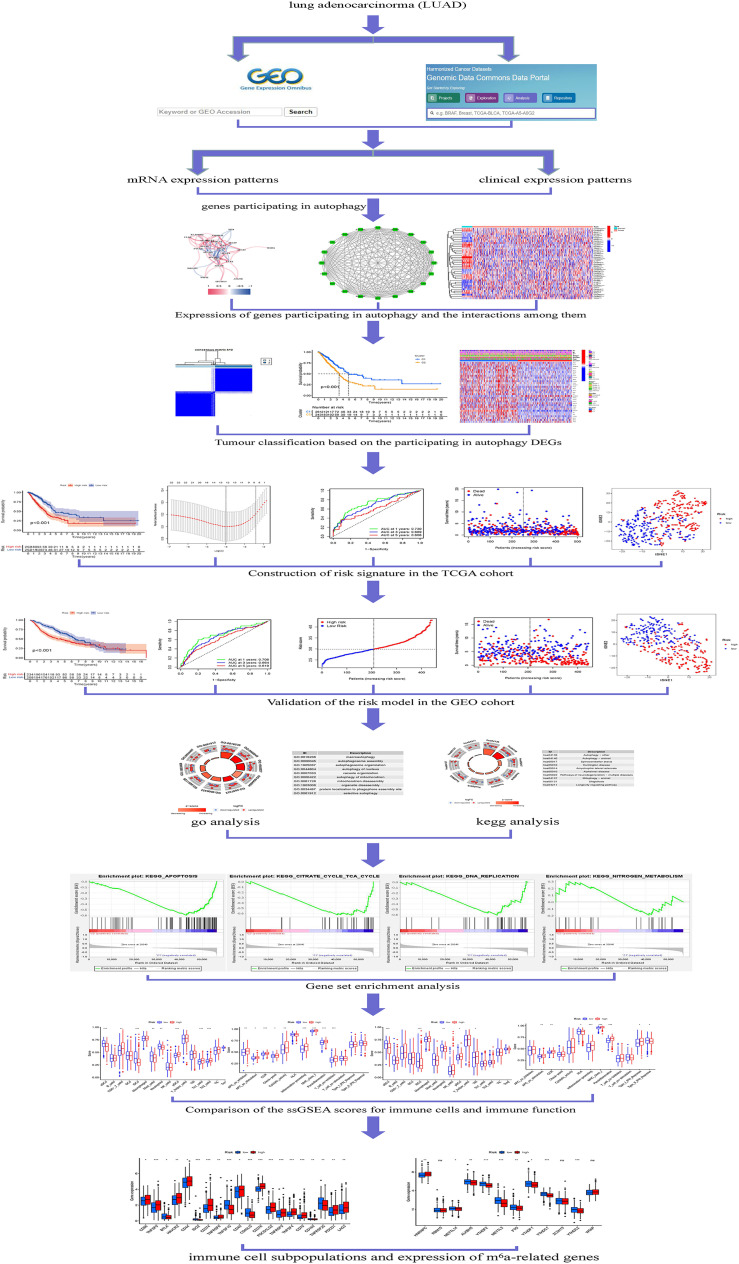
Framework based on an integration strategy of genes participating in autophagy. The data of LUAD patients were obtained from TCGA and GEO databases, and then the autophagy-related genes were matched to carry out difference analysis and risk model construction, respectively. TCGA dataset was used as the main body and GEO dataset was used to verify the model with good grouping, and genes participating in autophagy related to the prognosis of LUAD patients were obtained. Then, Gene Ontology (GO), Kyoto Encyclopedia of Genes and Genomes (KEGG), and gene set enrichment analysis (GSEA) were performed with multiple databases to obtain the functions related to genes participating in autophagy. Last, the immune cells and function were analyzed. *P < 0.05; **P < 0.01; ***P < 0.001.

Immune checkpoint-related gene (ICRG) profiles in LUAD patients may facilitate determining, evaluating, and predicting treatment responses (12, 13). Despite the very fact that there has been very little analysis on the link between genes participating in autophagy and LUAD, it is very essential to study the interaction between genes participating in autophagy, immunity, immunological checkpoints, and m^6^a with LUAD clinicopathological tumor options. At this time, the cause and mechanism of LUAD’s abnormal organic phenomenon and autophagy are unknown. Transcriptions of genes participating in autophagy alterations in LUAD patients are needed to perceive the genes participating in autophagy pathways that influence the prognosis of LUAD patients. In LUAD patients, ICRG profiles may be utilized to predict medical care response, quantify risk, and predict OS. Understanding, however, the impact of genes participating in autophagy on LUAD development may invent a biomarker that might be utilized as a therapeutic target The strategy of genes participating in autophagy is shown in **Figure 1**.

This study aimed to form a prognostic model for LUAD prognosis prediction by spotting genes participating in autophagy expression related to LUAD patient prognosis. By better understanding the invasion of genes participating in autophagy and their associated targets, the innovative LUAD therapeutic targets and pharmacologic approaches will be facilitated developing.

## Materials and Methods

### Datasets and Genes Participating in Autophagy

LUAD gene expression patterns and clinical data were collected from The Cancer Genome Atlas (TCGA) (14). In October 21, 2021, the expression patterns of 535 instances of LUAD and 59 cases of normal tissues were enrolled in TCGA. The Gene Expression Omnibus (GEO) was searched for micro data on mRNA expression. Series: GSE68465. Platform: GPL570. The GEO was used to maintain the expression patterns of 462 LUAD cases. **Table 1** summarized the clinical features of the patients. In addition, 139 genes participating in autophagy in total were identified from KEGG (https://www.kegg.jp/kegg/) (**Table S1**).

**Table 1:** The clinical characteristics of patients.

TCGA		GEO	
Variables	Number of samples	Variables	Number of samples
Gender		Gender	
Male/female	242/280	Male/female/NA	223/220/19
Age at diagnosis		Age at diagnosis	
≤65/>65/NA	241/262/19	≤65/>65	231/212
Stage		Stage	
I/II/III/IV/NA	279/124/85/26/8	I/II/III/IV/NA	Unknown
T		T	
T1/T2/T3/T4/NA	172/281/47/19/3	T1/T2/T3/T4	Unknown
M		M	
M0/M1/NA	353/25/144	M0/M1/NA	Unknown
N		N	
N0/N1/N2/N3/NA	335/98/75/2/12	N0/N1/N2/N3	Unknown

GEO, Gene Expression Omnibus; TCGA, The Cancer Genome Atlas; T, T stage; M, M stage; N, N stage.

### Annotation of Genes

Transcription data and human configuration files were matched and sorted by Perl to obtain the precise mRNA gene expression data. Using information from the ensemble database, the gene IDs were transformed into gene names. The R4.1.0 Limma was used to retrieve the genes participating in autophagy expression data.

### Identification of Participating in Autophagy Differentially Expressed Genes and Their Mutation Rate Analysis

False discovery rate (FDR) <0.05 and |log2FC| ≥0.585 were used to evaluate a significant difference in genes participating in autophagy expression. First, the functions of differential genes participating in autophagy that were both upregulated and downregulated [differentially expressed genes (DEGs)] were looked into. The genetic changes of these genes were investigated because of the significant clinical consequences of these genes participating in autophagy. DEG mutation rates were examined using Cbioportal (http://www.cbioportal.org/).

### Tumor Classification Based on the Differentially Expressed Genes

First, the prognosis-related genes participating in autophagy were classified into two groups: cluster 1 and cluster 2. Survminer was used to explore the survival of genes participating in autophagy subtypes, and survival was used to evaluate genes participating in autophagy predictive value. The pheatmap was used to construct a heatmap showing the differential expression of genes participating in autophagy in each cluster, and the relationship between genes participating in autophagy and clinicopathological features was examined. Limma was used to identify differences in the expression of target genes from the appropriate subtypes and tissue types. To explore the gene connection between LUAD target genes and prognostic genes participating in autophagy, Limma and corrplot were employed.

### Development of Genes Participating in Autophagy Prognostic Signature

The risk score of every LUAD patient was additionally assessed. The DEGs were split into two classes that supported the median score: low-risk and high-risk. Least absolute shrinkage and selection operator (LASSO) regression was shown to be related to the low- and high-risk classes. Following the image, the boldness interval and risk ratio were computed, and therefore the forest diagram was created. Survival curves for the two groups were generated and compared. To evaluate the accuracy of this model for predicting survival in LUAD, the time dependent receiver operating characteristic curve (timeROC) was used to provide a comparable receiver operating characteristics (ROC) curve. For the chance curve bestowed by the risk score, genes participating in autophagy risk and survival status were examined. The nursing independent prognostic study was carried out to confirm that this model was unaffected by different clinical factors. The relationship between clinical characteristics and risk prediction model was determined, also the relationship between 2 genes participating in autophagy patients. Analyses of risk and clinical relationships were distributed. Additionally, principal component analysis (PCA) and t-distributed stochastic neighbor embedding (T-SNE) were investigated to analyze whether the prognostic model might properly categorize patients into two risk teams. By desegregation of the prognosticative signals, a representation was developed to predict the 1-, 2-, and 3-year OS of LUAD patients.

### Functional Enrichment of the Differentially Expressed Genes Participating in Autophagy

The biological pathways associated with TCGA DEGs were then examined using GO. Biological processes (BP), molecular functions (MF), and cellular components (CC) controlled by the DEGs participating in autophagy were further investigated using R software, clusterProfiler, org.Hs.eg.db, enrichplot, and ggplot2 package based on KEGG data.

### Gene Set Enrichment Analysis and the Predictive Nomogram

GSEA was used to find related functions and pathway variations in several samples, and Perl was used to import information. The associated score and graphs were wont to verify whether the functions and routes within the numerous risk groups were dynamic. Every sample was classified as “H” or “L” depending on whether it had been a high-risk cluster of prognosis-related genes participating in autophagy.

### Comparison of the Immune Activity Between Subgroups

The analysis of single sample gene set enrichment analysis (ssGSEA) was flexible. The enrichment score of immune cells and immune-related activities in the two groups were examined in each TCGA and GEO cohort. Additionally, the connections between genes participating in autophagy, checkpoints, and m^6^a were investigated, since these genes participating in autophagy have significant therapeutic implications.

## Results

Twenty-five participating in autophagy DEGs similarly to 12 risk genes participating in autophagy were found. GSEA was used to uncover latent signaling pathways involved within the development and progression of LUAD, and LASSO regression was accustomed to build an appropriate prediction model.

### Differentially Expressed Genes Participating in Autophagy

**Figure 2:**
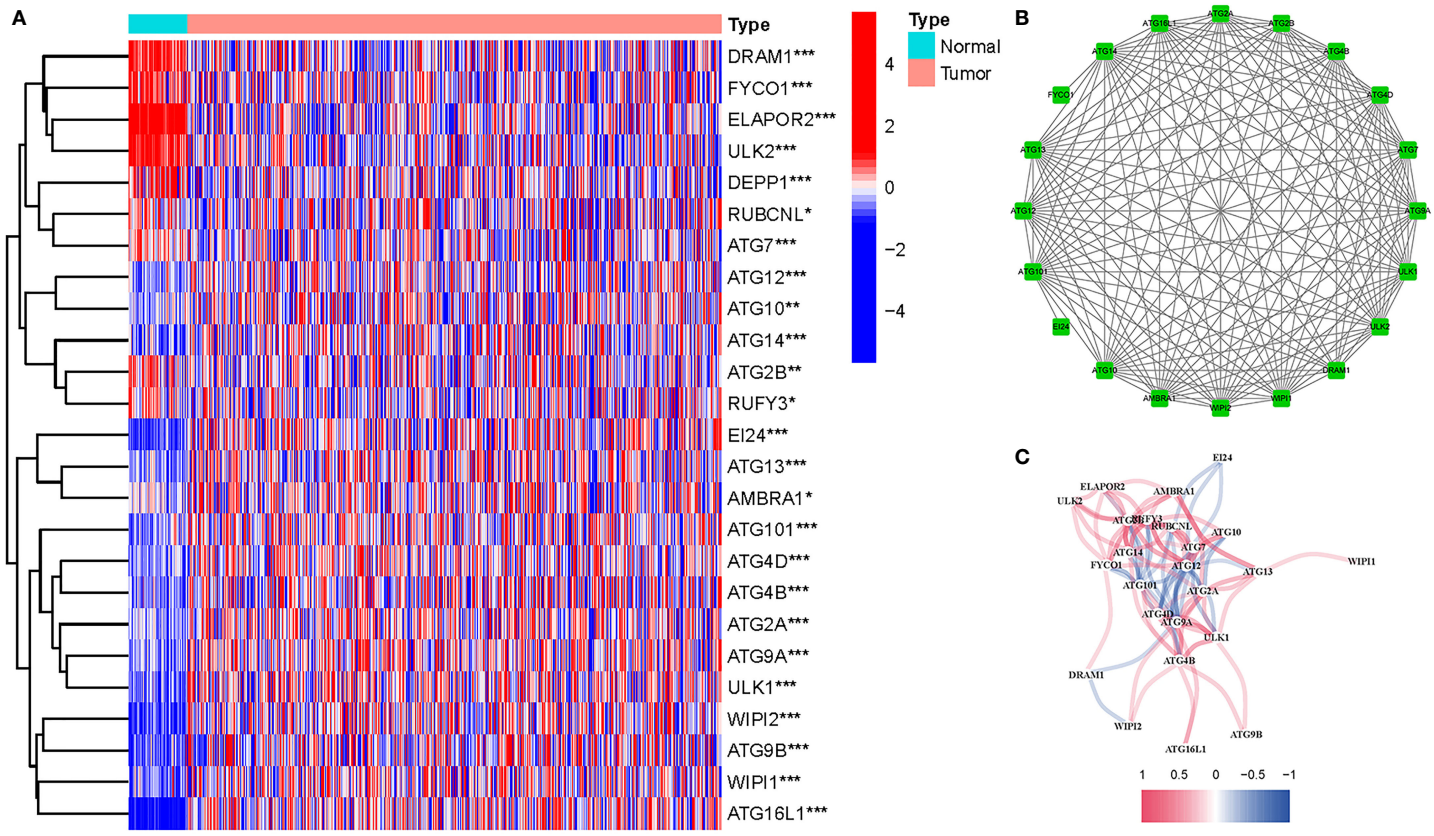
Expressions of the 25 genes participating in autophagy and the interactions among them. **(A)** Heatmap (green: low expression level; red: high expression level) of the genes participating in autophagy between the normal (N, brilliant blue) and the tumor tissues (T, red). P values were shown as *P < 0.05; **P < 0.01; ***P < 0.001. **(B)** PPI network showing the interactions of the genes participating in autophagy (interaction score = 0.4). **(C)** The correlation network of the genes participating in autophagy (red line: positive correlation; blue line: negative correlation. The depth of the colors reflects the strength of the relevance).

**Figure 3:**
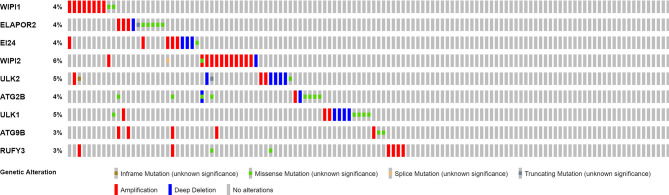
Mutations in genes participating in autophagy. A total of 10 genes have a mutation rate ≥3%.

Twenty-five DEGs associated with autophagy (17 upregulated, 8 downregulated; **Table S2**) were found (**Figure 2A**). To further examine the interactions of these genes participating in autophagy, a protein–protein interaction (PPI) analysis was conducted, and the results were given in **Figure 2B**. By putting the lowest required interaction value at 0.4, it was found that *ATG14*, *ATG101*, *AMBRA1*, *WIPI1*, *ATG10*, *ULK1*, *ATG7*, *ATG16L1*, *ULK2*, *ATG12*, and *ATG13* were hub genes (**Table S3**). These genes, which comprised all DEGs detected in normal and tumor tissues, may be found to be independent LUAD prognostic indicators. The correlation network, including all genes participating in autophagy, was depicted in **Figure 2C**. It was observed that truncating and missense mutations were the two most prevalent types of mutations (**Figure 3**). A total of nine genes showed a 3% mutation rate, with *WIPI2* being the most often changed (6%).

### Tumor Classification Based on the Differentially Expressed Genes

**Figure 4:**
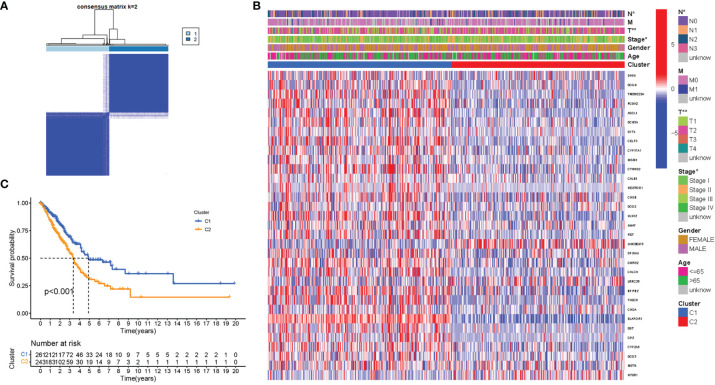
Tumor classification based on the participating in autophagy DEGs. **(A)** Here, 535 LUAD patients were grouped into two clusters according to the consensus clustering matrix (k = 2). **(B)** Heatmap. Heatmap and the clinicopathologic characters of the two clusters classified by these DEGs (T, N, and Stage are the degree of tumor differentiation. **(C)** Kaplan–Meier OS curves for the two clusters.

To investigate the links between ATG gene expression and LUAD subtypes, a consensus clustering analysis on all 535 LUAD patients were performed in TCGA dataset. It was discovered that when the clustering variable (k) was set to 2, the intragroup correlations were the highest and the intergroup correlations were the lowest, indicating that the 535 LUAD patients could be separated into two groups based on the genes participating in autophagy (**Figure 4A**). The gene expression profiles and clinical features were shown using a heatmap (**Figure 4B**). A survival study was undertaken to examine the predictive value of genes participating in autophagy utilizing PRG subtypes, and cluster 1 had a higher survival rate (P < 0.001), as shown in **Figure 4C**.

### Development of a Prognostic Gene Model in The Cancer Genome Atlas Cohort

**Figure 5:**
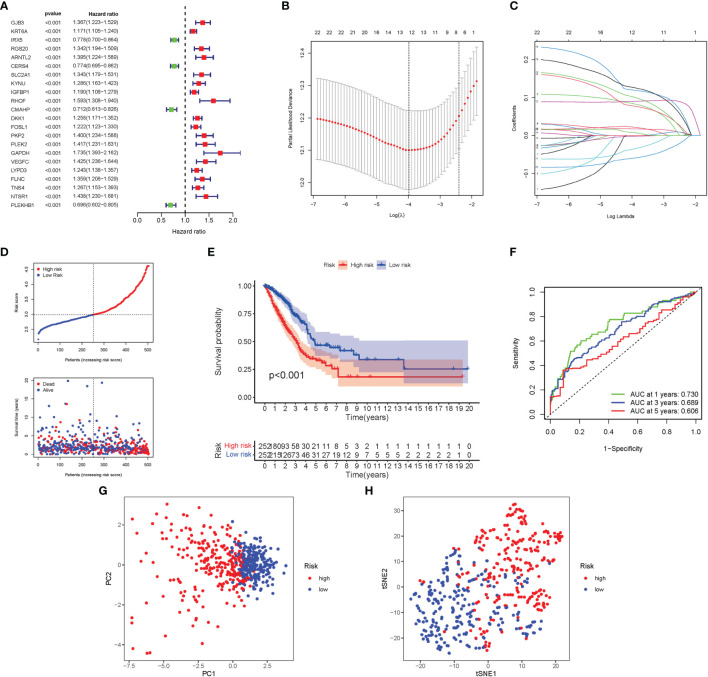
Construction of risk signature in the TCGA cohort. **(A)** A univariate Cox regression analysis of OS for each participating in autophagy gene and 22 genes with P < 0.01. **(B)** LASSO regression of the 22 OS-related genes. **(C)** Cross-validation for tuning the parameter selection in the LASSO regression. **(D)** The survival status for each patient (low-risk population: on the left side of the dotted line; high-risk population: on the right side of the dotted line). **(E)** Kaplan–Meier curves for the OS of patients in the high- and low-risk groups. **(F)** The AUC of the prediction of 1-, 2-, 3-year survival rate of LUAD. **(G)** PCA plot for LUADs based on the risk score. **(H)** t-SNE plot for LUADs based on the risk score.

Here, 22 major genes participating in autophagy were identified throughout the univariate Cox investigation. These genes participating in autophagy were discovered as independent LUAD prognostic markers (*GJB3*, *KRT6A*, *IRX5*, *RGS20*, *ARNTL2*, *CERS4*, *SLC2A1*, *KYNU*, *IGFBP1*, *RHOF*, *CMAHP*, *DKK1*, *FOSL1*, *PKP2*, *PLEK2*, *GAPDH*, *VEGFC*, *LYPD3*, *FLNC*, *TNS4*, *NTSR1*, *PLEKHB1*) (**Figure 5A**). A gene signature was created using the LASSO Cox regression analysis and the optimal value (**Figures 5B, C**). Employing a risk survival standing plot, it was tended to discover that a patient’s risk score was negatively connected to LUAD patients’ survival. The presence of high-risk PRG signatures were linked with a decreased chance of survival (P < 0.001; **Figure 5E**). For 1-, 2-, and 3-year survival rates, the AUC predictive value of the unique NRG signature was 0.730, 0.689, and 0.606, respectively (**Figure 5F**). PCA and t-SNE results showed that patients with varying risks were divided into two groups (**Figures 5G, H**).

### External Validation of the Risk Signature

**Figure 6:**
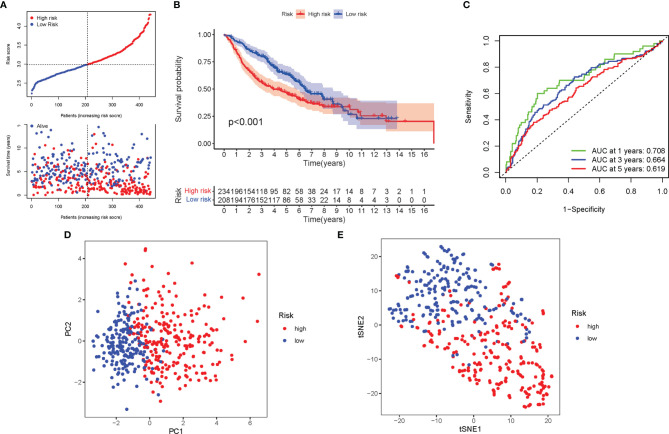
Validation of the risk model in the GEO cohort. **(A)** The survival status for each patient (low-risk population: on the left side of the dotted line; high-risk population: on the right side of the dotted line). **(B)** Kaplan–Meier curves for the OS of patients in the high- and low-risk groups. **(C)** The AUC of the for the prediction of 1-, 2-, 3-year survival rate of LUAD. **(D)** PCA plot for LUADs based on the risk score. **(E)** t-SNE plot for LUADs based on the risk score.

A total of 462 LUAD patients from a GEO cohort were enclosed within the validation group. It was tended to discover that a patient’s risk score was negatively associated with LUAD patients’ survival. Amazingly, similar with TCGA findings, the bulk of the novel genes participating in autophagy discovered during this study was adversely linked with this risk model (**Figure 6A**). High-risk PRG signatures were joined with a lower probability of survival (P < 0.001; **Figure 6B**). The AUC predictive value of the distinctive genes participating in autophagy signature was 0.708, 0.664, and 0.619 for 1-, 2-, and 3-year survival rates, respectively (**Figure 6C**). The results of PCA and t-SNE discovered that patients with varied risks were well divided into two groups (**Figures 6D, E**).

### Independent Prognostic Value of the Risk Model

**Figure 7:**
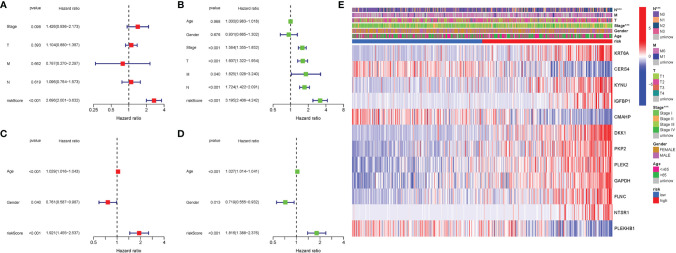
Univariate and multivariate Cox regression analyses. **(A)** Multivariate analysis for TCGA cohort. **(B)** Univariate analysis for TCGA cohort (T, M, N, Stage: the degree of tumor differentiation). **(C)** Multivariate analysis for the GEO cohort. **(D)** Univariate analysis for the GEO cohort (Age, Gender). **(E)** Heatmap (green: low expression; red: high expression) for the connections between clinicopathologic features and the risk groups. ***P < 0.001.

In TCGA cohort, Cox analysis demonstrated that the genes participating in autophagy signature [hazard ratio (HR): 2.696, 95% CI: 2.001–3.632] were primarily independent predictive variables for the OS of LUAD patients (**Figures 7A, B**). In the GEO cohort, Cox analysis demonstrated that the genes participating in autophagy signature (HR: 1.921, 95% CI: 1.455–2.537), age (HR: 1.029, 95% CI: 1.016–1.043), and gender (HR: 0.761, 95% CI: 0.587–0.987) were primarily independent predictive variables (**Figures 7C, D**). In addition, for TCGA cohort, a heatmap of clinical characteristics was constructed (**Figure 7E** and **Table S4**).

### Enrichment Analysis of Genes Participating in Autophagy

**Figure 8:**
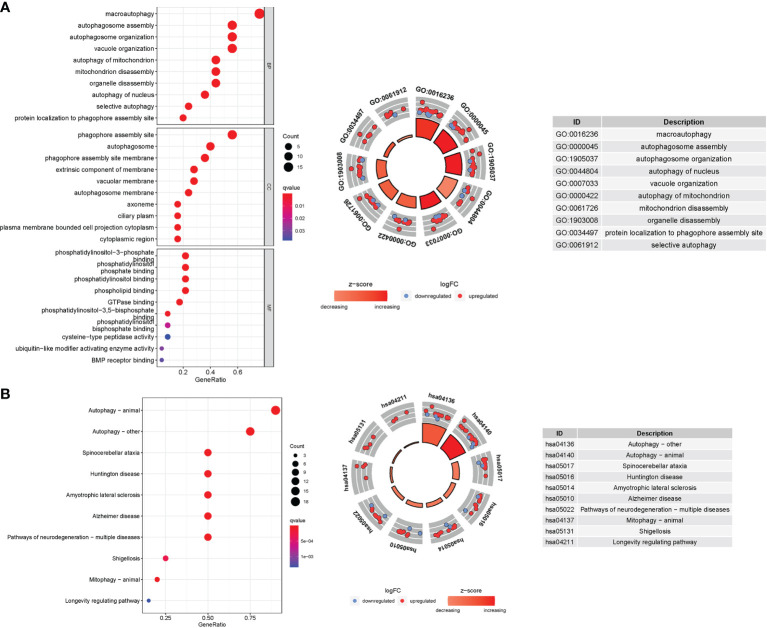
GO and KEGG analyses for genes participating in autophagy. **(A)** Bubble graph for GO enrichment (the bigger bubble means the more genes enriched, and the increasing depth of red means the differences were more obvious; q-value: the adjusted P value). The GO circle shows the scatter map of the logFC of the specified gene. **(B)** Barplot graph for KEGG pathways (the longer bar means the more genes enriched, and the increasing depth of red means the differences were more obvious). The KEGG circle shows the scatter map of the logFC of the specified gene. The higher the Z-score value indicated, the higher expression of the enriched pathway.

In TCGA cohort, 25 DEGs were discovered between the two groups. GO enrichment analysis revealed 91 core targets, including BP, MF, and CC. The MF mainly involved phospholipid binding (GO:0005543), phosphatidylinositol binding (GO:0035091), and protein serine kinase activity (GO:0106310). The CC mainly involved vacuolar membrane (GO:0005774), endocytic vesicle (GO:0030139), and extrinsic component of membrane (GO:0019898). The BP mainly involved response to extracellular stimulus (GO:0009991), cell growth (GO:0016049), and response to nutrient levels (GO:0031667). In addition, the main signaling pathways were identified by KEGG enrichment analysis, revealing that the overexpressed genes were mainly involved in pathways of neurodegeneration-multiple diseases (hsa05022), amyotrophic lateral sclerosis (hsa05014), autophagy-other (hsa04136), and autophagy-animal (hsa04140) (**Figure 8** and **Table S5**).

### Gene Set Enrichment Analyses

**Figure 9:**
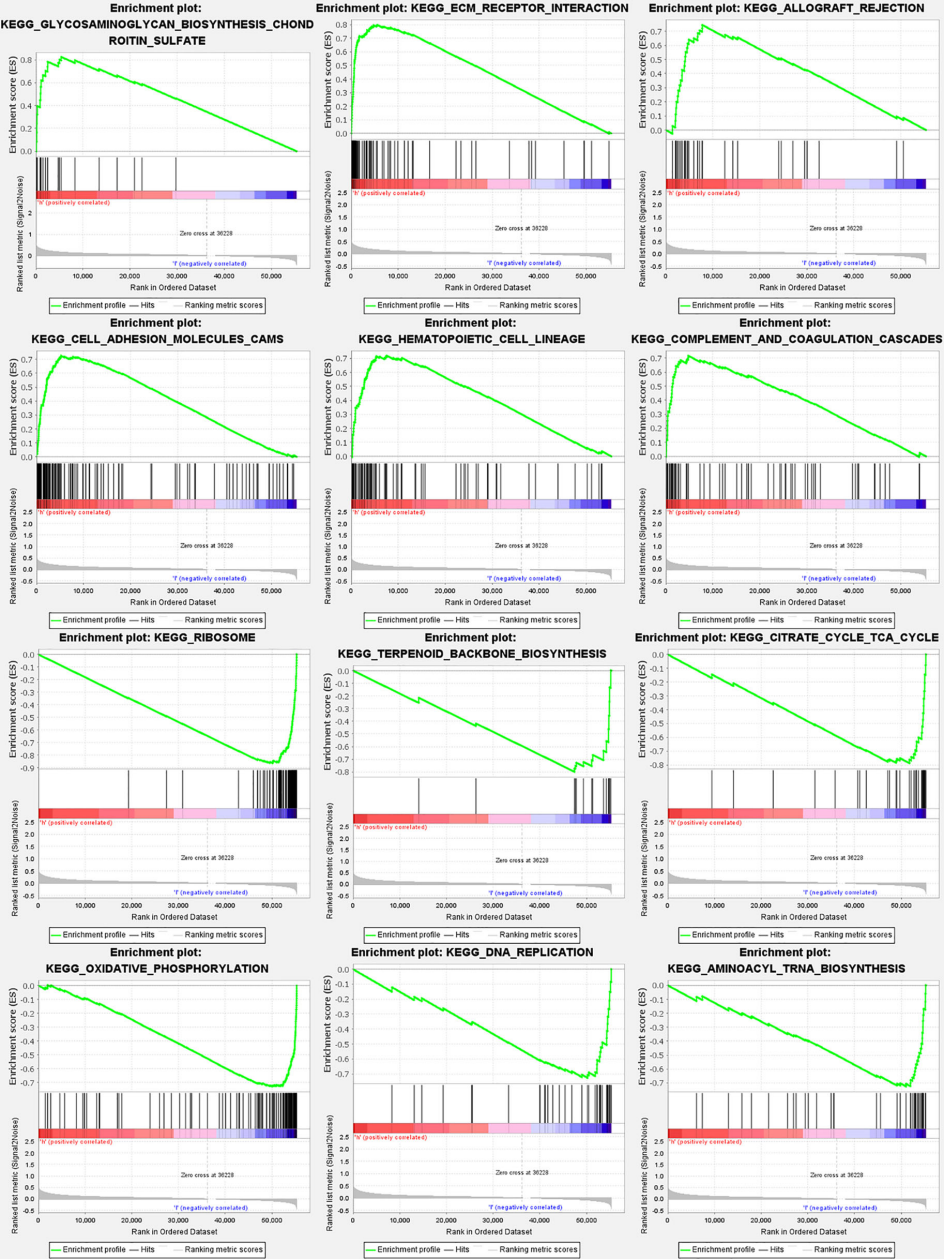
Gene set enrichment analyses for genes participating in autophagy. To clarify the difference of related function or pathway in different samples, the top 6 enriched functions or pathways of each cluster were listed. The most enriched pathway was the Hedgehog signaling pathway. Both FDR q-value and FWER P value were <0.05.

According to GSEA, the majority of genes participating in autophagy prognostic signature regulated immune and tumor-related pathways such as glycosaminoglycan biosynthesis chondroitin sulfate, extracellular matrix (ECM) receptor interaction, allograft rejection, Hedgehog (Hh) signaling pathway, and notch signaling pathway. The top 6 enriched functions or pathways for each cluster were shown in **Figure 9** and **Table S6**. The “hedgehog signaling pathway” was the most enriched, and some of the genes were positively correlated with H or L.

### Comparison of the Immune Activity Between Subgroups

**Figure 10:**
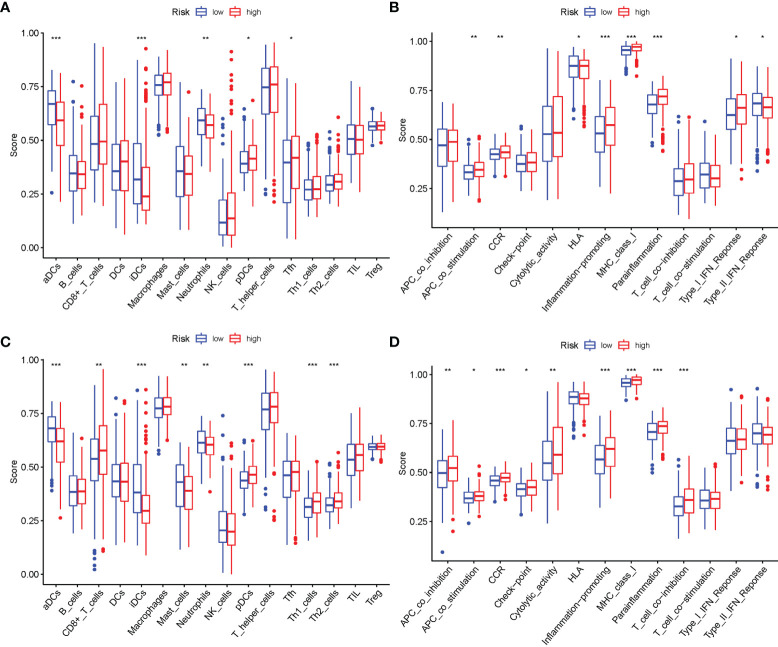
Comparison of the ssGSEA scores. **(A, B)** Comparison of the enrichment scores of 16 types of immune cells and 13 immune-related pathways between low-risk (green box) and high-risk (red box) group in TCGA cohort. **(C, D)** Comparison of the tumor immunity between low-risk (blue box) and high-risk (red box) group in the GEO cohort. *P < 0.05; **P < 0.01; ***P < 0.001.

The enrichment scores of 16 kinds of immune cells and the activity of 13 immune-related functions across the low- and high-risk groups in two cohorts (ssGSEA) were evaluated. *aDCs*, *iDCs*, *Neutrophils*, *pDCs*, and *Tfh* infiltrated at a greater rate in the high-risk subgroup (**Figure 10A**). APC costimulation, CCR, HLA, Inflammation-promoting, MHC class I, Parainflammation, Type I IFN Response, and Type II IFN Response were usually more significant in the high-risk group (**Figure 10B**). Similar findings were reached when examining the immunological state of the GEO cohort (**Figures 10C, D**).

### Analysis of the Correlation Between Genes Participating in Autophagy With Immune Checkpoints and m^6^a

**Figure 11:**
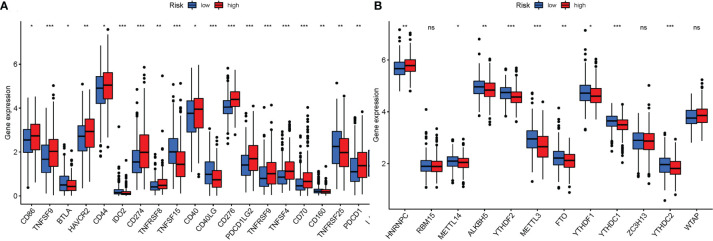
**(A)** Expression of immune checkpoints among high and low LUAD risk groups. **(B)** The expression of m^6^a-related genes between high and low LUAD risk groups. P values were shown as ns, not significant; *P < 0.05; **P < 0.01; ***P < 0.001.

Given the importance of checkpoint inhibitor-based immunotherapies, it was considered whether there were any changes in immune checkpoint expression between the two groups. The expression of *TNFSF9*, *IDO2*, *CD274*, *TNFSF15*, *CD40LG*, *CD276*, and other genes differed significantly between the two patient groups (**Figure 11A**). When PRG expression was examined, the relationship of m^6^a and genes participating in autophagy, *YTHDF2*, *METTL3*, *YTHDC1*, *YTHDC2*, *HNRNPC*, *METTL14*, *ALKBH5*, *FTO*, and *YTHDF1*, was substantially more significant in the high-risk group (**Figure 11B**).

## Discussion

LUAD therapy may be a significant therapeutic downside due to its severe condition and poor prognosis (15, 16). The molecular identification of diagnostic biomarkers and treatment targets for LUAD ought to be promoted at all times. Previous studies have shown that autophagy is concerned with the abnormal cell death related to LUAD (17, 18). Genes participating in autophagy can perform as a tumor suppressor, making them a promising cancer medical care approach (19). It is unknown, however, how it affects LUAD formation *via* dominant genes participating in autophagy. This study aimed to develop a predictive model by identifying genes participating in autophagy with expressions connected to LUAD patient prognosis.

Several RNAs in LUAD were found to be related to autophagy during this investigation. Following that, 25 DEGs coupled to autophagy were tended to know. Using the information on prognosis-related genes, the confidence interval and HR were calculated to examine their potential roles in LUAD. In a univariate Cox regression analysis, genes participating in autophagy were shown to be considerably associated with LUAD prognosis. The researchers identified 12 prognostic genes participating in autophagy that expressed differentially in different groups. Some genes participating in autophagy were discovered to be overexpressed at high risk. Others, on the other hand, were overexpressed in low risk (P < 0.05). It was tended to investigate a lot of into the role of genes participating in autophagy in LUAD. The prognostic significance of genes participating in autophagy was determined by employing a survival analysis supported gene subtypes. *KRT6A*, *KYNU*, *IGFBP1*, *DKK1*, *PKP2*, *PLEK2*, *GAPDH*, *FLNC*, and *NTSR1* were all overexpressed in insecure areas people, indicating that they may be related to the oncology process for LUAD patients; they seemed to be cancer-promoting genes. Our results on the above genes provide some insights for further research, but there is still no conclusive evidence that they are involved in the expression of specific transcription factors related to autophagy regulation, such as TFEB, HSF1, and FOXO3, which requires further investigation. *CERS4*, *CMAHP*, and *PLEKHB1* were found to be considerably expressed in low-risk people, suggesting that they are associated with a low risk in LUAD patients. The previously discovered genes participating in autophagy may be used as a therapeutic target for LUAD. Furthermore, within the LUAD analysis, genes participating in autophagy were coupled to patient outcomes. The OS and ROC analysis of the GSE68465 Kaplan–Meier curves indicated that a participating in autophagy signature might be an independent prognostic predictor. Solely a little amount of study has been conducted on the cistron alterations related to autophagy. Many more studies are needed to comprehend the NRG alteration and identification method and to validate the findings in this study.

Following that, KEGG analysis revealed that the genes were primarily concerned in amyotrophic lateral sclerosis, autophagy-other, and autophagy-animal. As a result, autophagy plays a vital role in LUAD. The Hh signal pathway was discovered to be the significant well-enriched route in GSEA. In invertebrates, the Hh pathway regulates sophisticated biological processes. As a result of the abnormal Hh pathway, activation is chargeable for carcinogenesis and cancer maintenance during a type of malignancies; addressing this provides a viable therapeutic opportunity (20). The Hh sign has been shown to suppress autophagy in normal and cancer cells from various tissue sources (21, 22) and has been shown in many investigations to activate autophagy. The Hh antagonist cyclopamine, for instance, reserved autophagy activation within the neuroblastoma cell line SHSY5Y (23). By bidirectionally regulation autophagy, the Hh sign influences a range of tissue origins. The findings listed above were under consideration. Genes participating in autophagy could influence LUAD cell migration and proliferation *via* modulating the Hh signaling pathway. Furthermore, methods in this study can accurately predict the survival of LUAD patients. An increase within the risk score is related to a rise in mortality and high-risk ratio. The conception could be utilized in a variety of therapeutic contexts. Genes participating in autophagy seem to be a potential biomarker for predicting LUAD patient outcomes, supporting literature findings and information.

Furthermore, the connections between genes participating in autophagy, immune cells, immunological function, immune checkpoints, and m^6^a were investigated and examined. Recent studies have discovered an affiliation between completely different cell death mechanisms and anticancer immunity (24, 25). Within the recent decade, immune checkpoint inhibitors (ICIs) have transformed cancer treatment. They are monoclonal antibodies, and those targeting programmed death 1 (PD-1) or PD-1 ligand (PD-L1) are accustomed treat LUAD (26). In ICI-resistant tumors, activating pyroptosis, ferroptosis, and necroptosis in conjunction with ICIs resulted in synergistically increased anticancer effectiveness (27, 28). By targeting Atg5 and Atg7 within the m^6^a-YTHDF2-dependent mechanism, FTO Alpha-Ketoglutarate Dependent Dioxygenase (FTO) plays a conservative and vital function in promoting autophagy and adipogenesis (29). A microscopic investigation of the connection between ICI, m^6^a, and pyrolysis has been conducted. Even though there has been very little analysis on genes participating in autophagy and LUAD, supported by the information presented above, it could be concluded that ARG alterations were associated with the onset and development of LUAD.

Although it is offered for theoretical underpinnings and analysis recommendations, this study has its limitations. First, it was tended to develop a validated genes participating in autophagy prediction signature exploitation of TCGA and GEO datasets. We tend to be unable to gather sufficient external information from different publicly offered sources to evaluate the model’s dependableness. Second, we tend to center on the signature’s 12 risk genes participating in autophagy in the early expression study. Despite this, no additional functional or mechanical analysis was conducted. Finally, no LUAD studies were conducted to substantiate the link between prognostic genes and shift. However, to completely grasp the facts declared above, we tend to conduct additional analysis.

## Conclusions

In conclusion, 12 expected genes participating in autophagy were found in LUAD patients as part of the autophagy regulation. It provides LUAD with a high degree of predictability. These findings contribute to an improved understanding of the connection between immunological, m^6^a, and autophagy, maybe paving the way for new therapeutic targets and prognostic indicators. It is hoped that the findings will aid in discovering genes participating in autophagy that promote LUAD growth, permitting us to learn additional concerning their potential role within the development and progression.

## Data Availability Statement

The original contributions presented in the study are included in the article/**Supplementary Material**. Further inquiries can be directed to the corresponding author.

## Ethics Statement

This article is not a clinical trial; hence, the ethics approval and consent to participation are not applicable.

## Author Contributions

ZX-W drafted and revised the article. XH and M-JC were in charge of data collection. P-DH conceived and designed this article, was in charge of syntax modification, and revised the article. ZG revised the article. All the authors have read and agreed to the final version of the article.

## Funding

This work was supported by the Health and Health Commission of Yunnan Province 2020 High-level TCM Reserve Talents Incubation Project (Yunwei TCM Development [2021] No. 1); The second round of construction project of The National Traditional Chinese Medicine School Heritage Studio of the State Administration of Traditional Chinese Medicine (National Traditional Chinese Medicine Teaching Letter [2019] 62); and Scientific and Technological Innovation Team of Acupuncture and Moxibustion Prevention and Treatment of Mental Disorders in Yunnan Colleges and Universities (No.: 2019YGC04).

## Conflict of Interest

The authors declare that the research was conducted in the absence of any commercial or financial relationships that could be construed as a potential conflict of interest.

## Publisher’s Note

All claims expressed in this article are solely those of the authors and do not necessarily represent those of their affiliated organizations, or those of the publisher, the editors and the reviewers. Any product that may be evaluated in this article, or claim that may be made by its manufacturer, is not guaranteed or endorsed by the publisher.

## Abbreviations

LUAD, lung adenocarcinoma; GO, Gene Ontology; AUC, area under the curve; MF, molecular functions; ICIs, immune checkpoint inhibitors; ROC, receiver operating characteristic; GSEA, gene set enrichment analysis; KEGG, Kyoto Encyclopedia of Genes and Genomes; TCGA, The Cancer Genome Atlas; ICRG, immune checkpoint-related gene; BP, biological processes; CC, cellular components; OS, overall survival; GEO, Gene Expression Omnibus; DEGs, differentially expressed genes.
