# Supplementary material for: Development and Validation of a Prognostic Index Based on Genes Participating in Autophagy in Patients With Lung Adenocarcinoma
Source: Front Oncol. 2022 Jan 25;11:799759. doi: 10.3389/fonc.2021.799759 (PMC8821527; doi:10.3389/fonc.2021.799759)
Supplement: Supplementary file 1 [file Table_1.doc]

Development and validation of prognostic index based on genes participating in autophagy in patients with lung adenocarcinoma

Zi-xuan Wu^1^, Xuyan Huang^1^, Min-jie Cai^2^, Pei-dong Huang^3^, Guan Zunhui^4^

**Supplementary appendix to the manuscript**

Contents of supplementary appendix

[Appendix 1 3](#__RefHeading___Toc17929)

[139 genes participating in autophagy 3](#__RefHeading___Toc10951)

[Appendix 2 5](#__RefHeading___Toc24238)

[DEGs linked to genes participating in autophagy 5](#__RefHeading___Toc11337)

[Table 2a. 25 DEGs linked to genes participating in autophagy. 5](#__RefHeading___Toc6264)

[Table 2b. 83 DEGs linked to genes participating in autophagy of TCGA. 7](#__RefHeading___Toc25109)

[Table 2c. 45 DEGs linked to genes participating in autophagy of GEO. 10](#__RefHeading___Toc29484)

[Appendix 3 12](#__RefHeading___Toc27853)

[hub genes analysis 12](#__RefHeading___Toc21800)

[Table 3. Hub genes. 12](#__RefHeading___Toc18575)

[Appendix 4 13](#__RefHeading___Toc9315)

[12 risk genes participating in autophagy 13](#__RefHeading___Toc31872)

[Appendix 5 14](#__RefHeading___Toc18508)

[GO and KEGG enrichment analysis 14](#__RefHeading___Toc25514)

[Table 5a. GO enrichment analysis. 14](#__RefHeading___Toc25534)

[Table 5b. KEGG enrichment analysis. 21](#__RefHeading___Toc16811)

[Appendix 6 22](#__RefHeading___Toc20316)

[gene set enrichment analyses (GSEA) 22](#__RefHeading___Toc10765)

[Table 6a. GSEA of high rish. 22](#__RefHeading___Toc24602)

[Table 6b. GSEA of low rish. 31](#__RefHeading___Toc14150)

# Appendix 1

**139 genes participating in autophagy**

**Table 1. 139 genes participating in autophagy**

| involved in autophagy | *KIAA1324* | *KIAA0226* | *KIAA0889* | *APG7-LIKE* | *APG4C* |
| --- | --- | --- | --- | --- | --- |
|  | *KIAA1324L* | *KIAA1632* | *APG3L* | *APG9L1* | *APG4D* |
|  | *KIAA0226L* | *KIAA0652* | *APG5L* | *APG12* | *APG4B* |
|  | *APG16L* | *APG12L* | *APG10L* | *APG5* | *APG4-C* |
|  | *APG3* | *APG5-LIKE* | *KIAA0831* | *APG3-LIKE* | *APG4-D* |
|  | *APG9L2* | *APG7L* | *PIG8* | *APG10* | *APG4A* |
|  | *DRAM1* | *RUBCNL* | *DRAM2* | *DCAF3* | *RUBCN* |
|  | *ZFYVE7* | *SOGA* | *NOS3AS* | *AMBRA1* | *EPG5* |
| participating in autophagy | *ATG3* | *ATG1B* | *ATG101* | *ATG18* | *ATG13* |
|  | *ATG4B* | *ATG7* | *ATG4C* | *ATG9A* | *ATG12* |
|  | *ATG16A* | *ATG4D* | *ATG5* | *ATG14L* | *ATG16B* |
|  | *ATG2B* | *ATG4A* | *ATG14* | *ATG1* | *Atg21* |
|  | *ATG10* | *ATG16L2* | *ATG18B* | *ATG1A* | *hATG1* |
|  | *hAPG5* | *ATG16L1* | *ATG9B* | *ATG18A* | *WIPI2* |
|  | *HAPG12* | *ATG2A* | *ATG16L* | *WIPI49* |  |
|  | *WIPI-2* | *WIPI1* | *FYCO1* | *mATG9* | *UNC51* |
| associated to autophagy | *ELAPOR1* | *C12orf44* | *C14orf103* | *Unc51.1* | *PC3-96* |
|  | *ELAPOR2* | *C20orf117* | *C13orf18* | *C10orf10* | *SOGA1* |
|  | *IAI3B* | *1A1-3B* | *pp12616* | *DEPP1* | *WDR80* |
|  | *AUTL4* | *AUTL3* | *AUTL2* | *CATC2* | *NBR1* |
|  | *ULK2* | *ULK1* | *MIG19* | *AUTL1* | *IDDSSA* |
|  | *EPG4* | *CORD21* | *EIG121* | *DRAM* | *CGI-50* |
|  | *FBR93* | *Fseg* | *RUBICON* | *TMEM77* | *SCAR15* |
|  | *SCAR31* | *HEEW1* | *EIG121L* | *ASP* | *DEPP* |
|  | *PACER* | *FIG* | *SCAR25* | *Unc51.2* | *TP53I8* |
|  | *WDR30* | *VICIS* | *IBD10* | *MGD3208* | *CTRCT18* |
|  | *RUFY3* | *M17S2* | *PRO180* | *PARATARG8* | *WWFQ154* |
|  | *SONE* | *GSA7* | *BARKOR* | *WDR94* | *EI24* |

# Appendix 2

## **DEGs linked to genes participating in autophagy**

**Table 2a. 25 DEGs linked to genes participating in autophagy.**

| gene | conMean | treatMean | logFC | pValue |
| --- | --- | --- | --- | --- |
| *DRAM1* | 70.38413915 | 36.69012502 | -0.939858538 | 6.75E-19 |
| *ATG101* | 7.251514576 | 9.489194898 | 0.388003335 | 4.76E-07 |
| *WIPI1* | 6.377516847 | 8.756137654 | 0.45729983 | 2.84E-09 |
| *ELAPOR2* | 9.412421763 | 2.504405684 | -1.910097687 | 2.53E-32 |
| *RUBCNL* | 0.981313583 | 0.882222462 | -0.153571737 | 0.036379425 |
| *EI24* | 26.90593797 | 38.09384436 | 0.501633288 | 9.58E-17 |
| *ATG4D* | 5.294451712 | 7.321440332 | 0.467646207 | 7.72E-09 |
| *ATG16L1* | 4.057061424 | 6.944260381 | 0.775385899 | 1.14E-29 |
| *ATG2A* | 5.164020034 | 6.768501679 | 0.390341907 | 8.64E-08 |
| *WIPI2* | 7.753084525 | 10.98641563 | 0.502878479 | 2.40E-18 |
| *DEPP1* | 124.1782624 | 40.68355282 | -1.609895072 | 3.63E-10 |
| *ULK2* | 4.156848797 | 2.449854359 | -0.762794286 | 4.02E-21 |
| *FYCO1* | 10.06638051 | 7.690935378 | -0.388314062 | 1.94E-08 |
| *ATG13* | 10.02245966 | 11.89660091 | 0.247312815 | 2.10E-07 |
| *AMBRA1* | 5.212034254 | 5.767772751 | 0.146167758 | 0.030540982 |
| *ATG4B* | 6.118858644 | 8.232013389 | 0.427982758 | 1.87E-07 |
| *ATG7* | 3.753252458 | 3.383901935 | -0.149453575 | 6.83E-05 |
| *ATG9A* | 8.000064542 | 10.18381458 | 0.348194512 | 1.69E-10 |
| *ATG12* | 3.930178949 | 4.85222323 | 0.304050921 | 7.65E-06 |
| *ATG14* | 4.497301915 | 6.192511187 | 0.461464831 | 7.02E-09 |
| *ATG2B* | 2.869490356 | 2.687537913 | -0.094509419 | 0.003712755 |
| *ATG10* | 1.380271763 | 1.661078792 | 0.267168159 | 0.003342346 |
| *ULK1* | 5.744462254 | 9.587117947 | 0.738925335 | 4.77E-13 |
| *ATG9B* | 0.302119863 | 1.188518625 | 1.975971569 | 1.04E-11 |
| *RUFY3* | 4.652712881 | 4.757422565 | 0.032108013 | 0.024137963 |

**Table 2b. 83 DEGs linked to genes participating in autophagy of TCGA.**

| gene | lowMean | highMean | logFC | pValue |
| --- | --- | --- | --- | --- |
| *TPX2* | 5.704623636 | 6.709933419 | 1.005309783 | 1.48E-19 |
| *SCNN1B* | 6.2817559 | 5.145626865 | -1.136129035 | 1.85E-20 |
| *FOLR1* | 8.633721858 | 7.44911225 | -1.184609609 | 1.68E-13 |
| *RNASE1* | 10.08062315 | 8.834595536 | -1.246027611 | 1.16E-18 |
| *SELENBP1* | 8.553852362 | 7.199309679 | -1.354542683 | 1.01E-28 |
| *GFRA3* | 4.607177576 | 3.141311622 | -1.465865954 | 1.74E-20 |
| *SLC47A1* | 4.382526414 | 3.105291291 | -1.277235123 | 4.67E-25 |
| *PLA2G1B* | 4.483042551 | 3.4759875 | -1.007055051 | 5.19E-12 |
| *GJB3* | 3.670373916 | 5.066448198 | 1.396074282 | 5.40E-34 |
| *KRT6A* | 3.492235631 | 5.388324529 | 1.896088897 | 1.56E-23 |
| *IRX5* | 5.79254649 | 4.585812808 | -1.206733682 | 5.55E-24 |
| *ARNTL2* | 4.08673987 | 5.305554173 | 1.218814303 | 9.48E-36 |
| *CACNA2D2* | 6.293254963 | 4.721830062 | -1.5714249 | 1.73E-26 |
| *PGC* | 8.212150723 | 5.753599645 | -2.458551078 | 3.38E-19 |
| *CYP2B7P* | 7.337722363 | 5.503136826 | -1.834585537 | 3.35E-25 |
| *SFTPC* | 8.25743312 | 6.93985637 | -1.31757675 | 6.64E-06 |
| *STEAP4* | 5.556273892 | 4.521862389 | -1.034411503 | 1.28E-11 |
| *CERS4* | 5.298766569 | 3.939860229 | -1.35890634 | 1.50E-27 |
| *TRIM29* | 3.716679431 | 4.735656171 | 1.01897674 | 3.53E-15 |
| *STEAP1* | 5.640437041 | 6.751032645 | 1.110595605 | 2.04E-21 |
| *SFTPD* | 8.383006502 | 6.975263666 | -1.407742836 | 1.91E-10 |
| *CDC20* | 5.270890865 | 6.350795983 | 1.079905118 | 3.30E-18 |
| *CAMK2N1* | 6.07547717 | 7.094093976 | 1.018616807 | 3.54E-22 |
| *SFTPB* | 12.14889671 | 9.880014089 | -2.268882625 | 3.80E-29 |
| *MMP1* | 5.462680782 | 6.953183064 | 1.490502281 | 5.98E-14 |
| *ZNF750* | 4.376142984 | 3.266356916 | -1.109786068 | 1.93E-16 |
| *SLC2A1* | 5.958867842 | 7.304771268 | 1.345903426 | 3.31E-42 |
| *WIF1* | 5.080149458 | 3.653938126 | -1.426211332 | 2.14E-10 |
| *MYBL2* | 4.919004979 | 6.010079014 | 1.091074035 | 2.05E-16 |
| *PARM1* | 7.504850746 | 6.374071858 | -1.130778887 | 4.16E-24 |
| *CRLF1* | 5.900433931 | 4.416127851 | -1.484306079 | 2.31E-09 |
| *C4BPA* | 8.399094563 | 7.18667161 | -1.212422953 | 1.40E-10 |
| *EREG* | 2.976262971 | 4.060414265 | 1.084151294 | 3.20E-16 |
| *PITX1* | 3.002126058 | 4.099493653 | 1.097367595 | 1.54E-15 |
| *TCN1* | 4.076418888 | 5.403813361 | 1.327394472 | 4.85E-13 |
| *FSCN1* | 5.553165089 | 6.667075299 | 1.113910209 | 2.60E-24 |
| *MAOA* | 7.204955028 | 6.200361488 | -1.00459354 | 5.64E-19 |
| *TMPRSS11E* | 3.390834153 | 4.449883047 | 1.059048894 | 2.99E-10 |
| *MALL* | 7.204339529 | 6.181584955 | -1.022754573 | 4.39E-18 |
| *ABCC2* | 2.3823034 | 3.434954747 | 1.052651346 | 4.92E-08 |
| *CDA* | 3.832453666 | 5.374465829 | 1.542012162 | 2.63E-18 |
| *ABCA3* | 7.226499699 | 5.947205692 | -1.279294006 | 1.97E-17 |
| *LMO3* | 7.125249142 | 5.861325629 | -1.263923513 | 7.43E-21 |
| *GPR87* | 3.444659429 | 4.967709936 | 1.523050507 | 3.04E-22 |
| *CYP4B1* | 6.726176833 | 5.104621169 | -1.621555664 | 1.03E-14 |
| *SERPINB5* | 2.569374906 | 4.303884231 | 1.734509325 | 4.76E-24 |
| *TMPRSS2* | 6.42484465 | 5.182772299 | -1.242072351 | 3.31E-23 |
| *PSCA* | 3.101010575 | 4.369434755 | 1.26842418 | 9.98E-14 |
| *AQP3* | 9.160616078 | 8.008509578 | -1.1521065 | 2.94E-14 |
| *CMAHP* | 5.868256866 | 4.861046485 | -1.007210381 | 2.31E-26 |
| *TFF1* | 4.466609624 | 5.742890435 | 1.276280811 | 6.17E-05 |
| *SLC22A3* | 5.019705501 | 3.900852689 | -1.118852812 | 8.63E-15 |
| *DKK1* | 3.75840058 | 5.634437213 | 1.876036633 | 6.35E-35 |
| *FOSL1* | 3.671103761 | 5.160150243 | 1.489046482 | 6.96E-27 |
| *PKP2* | 3.046435982 | 4.180145251 | 1.13370927 | 1.22E-34 |
| *PLEK2* | 5.18488912 | 6.275924235 | 1.091035115 | 3.30E-33 |
| *NKX2-1* | 8.264711826 | 6.440594865 | -1.82411696 | 3.29E-37 |
| *WFDC2* | 9.944905155 | 8.873971266 | -1.070933889 | 4.48E-14 |
| *AQP4* | 4.995330098 | 3.982529157 | -1.012800942 | 4.85E-12 |
| *S100A9* | 8.212923852 | 9.433434953 | 1.2205111 | 1.10E-11 |
| *HOPX* | 8.6693765 | 7.282129262 | -1.387247238 | 2.70E-18 |
| *PCP4* | 5.379492823 | 4.357740618 | -1.021752205 | 3.04E-07 |
| *SCTR* | 4.618629624 | 3.577178792 | -1.041450833 | 7.90E-13 |
| *SLC34A2* | 9.984986608 | 8.798083965 | -1.186902643 | 1.06E-13 |
| *PLAU* | 6.896156699 | 7.901098376 | 1.004941677 | 1.22E-15 |
| *KRT16* | 2.29095018 | 3.874041113 | 1.583090934 | 7.26E-28 |
| *COL11A1* | 4.618418937 | 5.696063335 | 1.077644398 | 3.34E-10 |
| *CDC45* | 3.203932142 | 4.291037195 | 1.087105052 | 1.17E-17 |
| *GGTLC1* | 6.249204721 | 4.943438782 | -1.305765939 | 6.37E-23 |
| *CDH3* | 5.764817951 | 6.792988768 | 1.028170817 | 6.84E-11 |
| *ARL14* | 1.811906907 | 3.050790986 | 1.238884079 | 1.91E-22 |
| *S100A8* | 5.953636028 | 7.017324755 | 1.063688727 | 3.54E-11 |
| *SLCO4A1* | 4.005787471 | 5.053685078 | 1.047897607 | 7.15E-22 |
| *CRYM* | 5.986252403 | 4.907293939 | -1.078958464 | 6.39E-16 |
| *PIGR* | 7.761195394 | 6.47508662 | -1.286108774 | 1.64E-11 |
| *LYPD3* | 3.768220291 | 5.147641012 | 1.379420722 | 4.24E-26 |
| *LAMC2* | 5.723398577 | 7.095885531 | 1.372486953 | 2.64E-31 |
| *TNS4* | 2.85775766 | 4.264738872 | 1.406981212 | 1.49E-25 |
| *TMEM59L* | 4.297243324 | 3.26792929 | -1.029314034 | 2.03E-13 |
| *FOXM1* | 4.660744695 | 5.663140605 | 1.002395909 | 1.93E-18 |
| *C1orf116* | 7.114076302 | 5.747590716 | -1.366485586 | 2.19E-16 |
| *AKR1B10* | 4.257406232 | 5.60711213 | 1.349705898 | 8.48E-10 |
| *LPCAT1* | 9.421518229 | 8.410574778 | -1.010943451 | 1.78E-19 |

**Table 2c. 45 DEGs linked to genes participating in autophagy of GEO.**

| gene | lowMean | highMean | logFC | pValue |
| --- | --- | --- | --- | --- |
| *FOLR1* | 8.579960263 | 7.541973931 | -1.037986332 | 1.22E-08 |
| *GFRA3* | 4.516093451 | 3.385474893 | -1.130618558 | 4.16E-08 |
| *GJB3* | 3.729647341 | 5.05371203 | 1.324064689 | 4.06E-31 |
| *MUC5AC* | 3.621142597 | 4.815389491 | 1.194246894 | 8.89E-09 |
| *KRT6A* | 3.371170508 | 5.497694541 | 2.126524033 | 9.29E-25 |
| *IRX5* | 5.740652328 | 4.712852497 | -1.027799832 | 1.05E-14 |
| *ARNTL2* | 4.094126288 | 5.170408308 | 1.07628202 | 1.21E-27 |
| *CACNA2D2* | 6.304736494 | 4.911033739 | -1.393702755 | 3.95E-19 |
| *PGC* | 7.567176099 | 6.529663935 | -1.037512164 | 0.00010316 |
| *CYP2B7P* | 7.378700039 | 5.822349195 | -1.556350845 | 6.97E-17 |
| *SFTPC* | 8.437573185 | 7.270720692 | -1.166852493 | 3.33E-05 |
| *CERS4* | 5.251319325 | 4.05970874 | -1.191610585 | 1.35E-17 |
| *TRIM29* | 3.619749356 | 4.728925894 | 1.109176539 | 2.23E-15 |
| *SFTPB* | 12.00267886 | 10.72018274 | -1.282496121 | 7.76E-15 |
| *MMP1* | 5.370689506 | 6.908571149 | 1.537881644 | 3.77E-13 |
| *SLC2A1* | 6.063890353 | 7.113144946 | 1.049254593 | 3.47E-23 |
| *TFF2* | 2.377056463 | 3.460571823 | 1.08351536 | 1.17E-08 |
| *KYNU* | 3.957313383 | 4.987137596 | 1.029824213 | 4.51E-16 |
| *TCN1* | 4.183742959 | 5.330365614 | 1.146622655 | 1.21E-09 |
| *GPX2* | 5.574513974 | 6.585251102 | 1.010737128 | 0.00085277 |
| *CDA* | 3.915507856 | 5.172527322 | 1.257019465 | 7.64E-12 |
| *SCGB1A1* | 7.478921289 | 6.312825685 | -1.166095604 | 0.000188318 |
| *IL1R2* | 3.037101741 | 4.212430323 | 1.175328582 | 3.83E-24 |
| *CYP4B1* | 6.809702859 | 5.394560741 | -1.415142118 | 3.29E-11 |
| *SERPINB5* | 2.555369163 | 4.111770722 | 1.556401559 | 7.71E-13 |
| *PSCA* | 3.094071539 | 4.332824751 | 1.238753211 | 1.63E-10 |
| *HSD17B6* | 5.748832385 | 4.593264061 | -1.155568324 | 2.17E-14 |
| *TFF1* | 3.999069021 | 5.626250402 | 1.627181382 | 5.34E-11 |
| *FGA* | 4.528340032 | 5.558433588 | 1.030093556 | 6.10E-06 |
| *MUC5B* | 4.539070597 | 5.967672445 | 1.428601849 | 2.59E-08 |
| *DKK1* | 3.705562215 | 5.370810093 | 1.665247877 | 1.33E-26 |
| *NKX2-1* | 8.206515859 | 6.983208991 | -1.223306868 | 2.74E-23 |
| *S100A9* | 8.317318204 | 9.577581774 | 1.26026357 | 7.42E-13 |
| *TOX3* | 5.786398693 | 4.610523182 | -1.175875511 | 1.71E-10 |
| *KRT16* | 2.557306761 | 3.655285278 | 1.097978516 | 3.71E-10 |
| *GGTLC1* | 6.232601836 | 5.142720513 | -1.089881322 | 2.61E-17 |
| *ARL14* | 1.83069917 | 3.041927356 | 1.211228187 | 2.61E-15 |
| *S100P* | 7.101511069 | 8.895377094 | 1.793866025 | 9.61E-13 |
| *PI3* | 3.638080469 | 4.777274218 | 1.139193749 | 1.16E-14 |
| *CRYM* | 6.04944786 | 5.040177837 | -1.009270024 | 1.25E-13 |
| *ANXA10* | 2.512276483 | 3.54875701 | 1.036480527 | 2.13E-08 |
| *LYPD3* | 3.822453638 | 4.910638641 | 1.088185003 | 1.53E-13 |
| *LAMC2* | 5.884890558 | 7.037900136 | 1.153009578 | 7.87E-22 |
| *AKR1B10* | 4.131749317 | 5.642086611 | 1.510337295 | 3.31E-10 |
| *PRSS2* | 3.729262857 | 4.768113503 | 1.038850646 | 2.98E-10 |

# Appendix 3

## **hub genes analysis**

**Table 3. Hub genes.**

| name | Betweenness | Closeness | Degree | Network |
| --- | --- | --- | --- | --- |
| *ATG14* | 36.74038462 | 0.95 | 18 | 16.94117647 |
| *ATG101* | 36.74038462 | 0.95 | 18 | 16.94117647 |
| *AMBRA1* | 0.740384615 | 0.904761905 | 17 | 17 |
| *WIPI1* | 0.740384615 | 0.904761905 | 17 | 17 |
| *ATG10* | 0.740384615 | 0.904761905 | 17 | 17 |
| *ULK1* | 0.740384615 | 0.904761905 | 17 | 17 |
| *ATG7* | 0.740384615 | 0.904761905 | 17 | 17 |
| *ATG16L1* | 0.740384615 | 0.904761905 | 17 | 17 |
| *ULK2* | 0.740384615 | 0.904761905 | 17 | 17 |
| *ATG12* | 0.740384615 | 0.904761905 | 17 | 17 |
| *ATG13* | 0.740384615 | 0.904761905 | 17 | 17 |
| *WIPI2* | 0.125 | 0.863636364 | 16 | 15.86666667 |
| *ATG4D* | 0.615384615 | 0.863636364 | 16 | 15.65 |
| *ATG2B* | 0.125 | 0.863636364 | 16 | 15.86666667 |
| *ATG2A* | 0.125 | 0.863636364 | 16 | 15.86666667 |
| *ATG4B* | 0.615384615 | 0.863636364 | 16 | 15.65 |
| *ATG9A* | 0.125 | 0.863636364 | 16 | 15.86666667 |
| *DRAM1* | 0.125 | 0.76 | 13 | 12.83333333 |
| *EI24* | 0 | 0.5 | 1 | 0 |
| *FYCO1* | 0 | 0.5 | 1 | 0 |

# Appendix 4

**12 risk genes participating in autophagy**

**Table 4. 12 risk genes participating in autophagy.**

| id | TCGA-50-5930 | TCGA-75-5125 | TCGA-L9-A7SV | TCGA-50-5051 |
| --- | --- | --- | --- | --- |
| *KRT6A* | 7.580136 | 3.3094781 | 2.5833748 | 2.829608 |
| *CERS4* | 4.1256474 | 3.8602583 | 6.4486642 | 6.74673 |
| *KYNU* | 5.6676349 | 3.8839723 | 3.7118672 | 4.991777 |
| *IGFBP1* | 5.0313801 | 2.3008074 | 2.214668 | 2.327517 |
| *CMAHP* | 4.8350667 | 5.2993144 | 5.4975665 | 5.368583 |
| *DKK1* | 9.6532432 | 3.1741762 | 3.1354435 | 3.344571 |
| *PKP2* | 5.0105963 | 4.7103632 | 2.2835269 | 2.79089 |
| *PLEK2* | 5.7941028 | 5.873331 | 6.0234752 | 5.963281 |
| *GAPDH* | 11.2759204 | 12.0691642 | 9.9571848 | 11.43474 |
| *FLNC* | 4.5073401 | 4.3692829 | 3.372599 | 3.65454 |
| *NTSR1* | 1.8469593 | 1.8869642 | 1.9231159 | 1.866445 |
| *PLEKHB1* | 5.7515606 | 5.4612988 | 6.5182176 | 6.758681 |

# Appendix 5

## **GO and KEGG enrichment analysis**

**Table 5a. GO enrichment analysis.**

| ONTOLOGY | ID | Description | BgRatio | pvalue | qvalue |
| --- | --- | --- | --- | --- | --- |
| BP | GO:0016236 | macroautophagy | 311/18862 | 1.25E-29 | 2.31E-27 |
| BP | GO:0000045 | autophagosome assembly | 96/18862 | 1.24E-26 | 1.15E-24 |
| BP | GO:1905037 | autophagosome organization | 99/18862 | 1.96E-26 | 1.21E-24 |
| BP | GO:0044804 | autophagy of nucleus | 14/18862 | 4.90E-24 | 2.27E-22 |
| BP | GO:0007033 | vacuole organization | 176/18862 | 9.14E-23 | 3.39E-21 |
| BP | GO:0000422 | autophagy of mitochondrion | 75/18862 | 7.78E-21 | 2.06E-19 |
| BP | GO:0061726 | mitochondrion disassembly | 75/18862 | 7.78E-21 | 2.06E-19 |
| BP | GO:1903008 | organelle disassembly | 107/18862 | 4.82E-19 | 1.12E-17 |
| BP | GO:0034497 | protein localization to phagophore assembly site | 11/18862 | 1.23E-12 | 2.53E-11 |
| BP | GO:0061912 | selective autophagy | 62/18862 | 1.66E-10 | 3.07E-09 |
| BP | GO:0042594 | response to starvation | 196/18862 | 4.82E-09 | 8.12E-08 |
| BP | GO:0010506 | regulation of autophagy | 328/18862 | 6.42E-09 | 9.91E-08 |
| BP | GO:0006497 | protein lipidation | 90/18862 | 1.09E-07 | 1.55E-06 |
| BP | GO:0042158 | lipoprotein biosynthetic process | 94/18862 | 1.36E-07 | 1.79E-06 |
| BP | GO:0031669 | cellular response to nutrient levels | 210/18862 | 2.63E-07 | 3.25E-06 |
| BP | GO:0031668 | cellular response to extracellular stimulus | 235/18862 | 5.10E-07 | 5.90E-06 |
| BP | GO:0018410 | C-terminal protein amino acid modification | 14/18862 | 7.41E-07 | 7.89E-06 |
| BP | GO:0042157 | lipoprotein metabolic process | 133/18862 | 7.67E-07 | 7.89E-06 |
| BP | GO:0031667 | response to nutrient levels | 451/18862 | 1.41E-06 | 1.38E-05 |
| BP | GO:0009267 | cellular response to starvation | 157/18862 | 1.74E-06 | 1.61E-05 |
| BP | GO:0009991 | response to extracellular stimulus | 477/18862 | 2.05E-06 | 1.81E-05 |
| BP | GO:0071496 | cellular response to external stimulus | 303/18862 | 2.24E-06 | 1.86E-05 |
| BP | GO:0098780 | response to mitochondrial depolarisation | 20/18862 | 2.31E-06 | 1.86E-05 |
| BP | GO:0000423 | mitophagy | 24/18862 | 4.09E-06 | 3.03E-05 |
| BP | GO:0044068 | modulation by symbiont of host cellular process | 24/18862 | 4.09E-06 | 3.03E-05 |
| BP | GO:0044003 | modulation by symbiont of host process | 37/18862 | 1.55E-05 | 0.000110535 |
| BP | GO:0097352 | autophagosome maturation | 41/18862 | 2.12E-05 | 0.000145521 |
| BP | GO:0016241 | regulation of macroautophagy | 160/18862 | 5.49E-05 | 0.00036322 |
| BP | GO:0061709 | reticulophagy | 12/18862 | 0.000110411 | 0.000705346 |
| BP | GO:0009896 | positive regulation of catabolic process | 450/18862 | 0.000270641 | 0.001671328 |
| BP | GO:0051817 | modulation of process of other organism involved in symbiotic interaction | 98/18862 | 0.000287884 | 0.001720463 |
| BP | GO:0048675 | axon extension | 114/18862 | 0.000448827 | 0.00259847 |
| BP | GO:0010508 | positive regulation of autophagy | 120/18862 | 0.000521448 | 0.002927428 |
| BP | GO:0035821 | modulation of process of other organism | 123/18862 | 0.000560419 | 0.003053678 |
| BP | GO:0043552 | positive regulation of phosphatidylinositol 3-kinase activity | 33/18862 | 0.000868346 | 0.004596358 |
| BP | GO:0090218 | positive regulation of lipid kinase activity | 37/18862 | 0.00109175 | 0.005618362 |
| BP | GO:1990138 | neuron projection extension | 164/18862 | 0.00128962 | 0.006457271 |
| BP | GO:0043551 | regulation of phosphatidylinositol 3-kinase activity | 57/18862 | 0.002574168 | 0.012549961 |
| BP | GO:0016239 | positive regulation of macroautophagy | 60/18862 | 0.002847878 | 0.01352838 |
| BP | GO:0051701 | biological process involved in interaction with host | 219/18862 | 0.002940616 | 0.013619696 |
| BP | GO:0048588 | developmental cell growth | 222/18862 | 0.003055695 | 0.013691152 |
| BP | GO:0060560 | developmental growth involved in morphogenesis | 225/18862 | 0.003173523 | 0.013691152 |
| BP | GO:0016049 | cell growth | 470/18862 | 0.003177748 | 0.013691152 |
| BP | GO:0043550 | regulation of lipid kinase activity | 69/18862 | 0.003747222 | 0.015777778 |
| BP | GO:0032984 | protein-containing complex disassembly | 328/18862 | 0.00902594 | 0.037159424 |
| BP | GO:0018215 | protein phosphopantetheinylation | 331/18862 | 0.009252473 | 0.037263966 |
| BP | GO:0043687 | post-translational protein modification | 361/18862 | 0.011702975 | 0.046130429 |
| CC | GO:0000407 | phagophore assembly site | 31/19520 | 8.80E-35 | 2.41E-33 |
| CC | GO:0034045 | phagophore assembly site membrane | 15/19520 | 8.99E-24 | 1.23E-22 |
| CC | GO:0005776 | autophagosome | 98/19520 | 1.95E-17 | 1.78E-16 |
| CC | GO:0000421 | autophagosome membrane | 40/19520 | 8.61E-12 | 5.89E-11 |
| CC | GO:0019898 | extrinsic component of membrane | 304/19520 | 7.84E-08 | 4.29E-07 |
| CC | GO:0005774 | vacuolar membrane | 431/19520 | 8.31E-07 | 3.79E-06 |
| CC | GO:0005930 | axoneme | 128/19520 | 2.01E-05 | 7.29E-05 |
| CC | GO:0097014 | ciliary plasm | 130/19520 | 2.13E-05 | 7.29E-05 |
| CC | GO:0032838 | plasma membrane bounded cell projection cytoplasm | 216/19520 | 0.000153691 | 0.000467365 |
| CC | GO:0099568 | cytoplasmic region | 258/19520 | 0.000303087 | 0.000829501 |
| CC | GO:0045335 | phagocytic vesicle | 136/19520 | 0.000680036 | 0.001691956 |
| CC | GO:0031312 | extrinsic component of organelle membrane | 52/19520 | 0.002007783 | 0.004579155 |
| CC | GO:0061695 | transferase complex, transferring phosphorus-containing groups | 253/19520 | 0.004007888 | 0.008437659 |
| CC | GO:1902554 | serine/threonine protein kinase complex | 89/19520 | 0.005760228 | 0.011260596 |
| CC | GO:0005811 | lipid droplet | 95/19520 | 0.00653705 | 0.011729362 |
| CC | GO:0030139 | endocytic vesicle | 307/19520 | 0.006857165 | 0.011729362 |
| CC | GO:1902911 | protein kinase complex | 104/19520 | 0.00778659 | 0.012535687 |
| CC | GO:0032045 | guanyl-nucleotide exchange factor complex | 13/19520 | 0.016527288 | 0.02512921 |
| CC | GO:0055037 | recycling endosome | 190/19520 | 0.024411957 | 0.033386259 |
| CC | GO:0044233 | mitochondria-associated endoplasmic reticulum membrane | 20/19520 | 0.025317655 | 0.033386259 |
| CC | GO:0005741 | mitochondrial outer membrane | 195/19520 | 0.025617533 | 0.033386259 |
| CC | GO:0031968 | organelle outer membrane | 220/19520 | 0.03199964 | 0.038714374 |
| CC | GO:0019867 | outer membrane | 222/19520 | 0.032534965 | 0.038714374 |
| CC | GO:0005942 | phosphatidylinositol 3-kinase complex | 29/19520 | 0.036508773 | 0.041632811 |
| CC | GO:0005802 | trans-Golgi network | 251/19520 | 0.040688121 | 0.044542785 |
| CC | GO:0005770 | late endosome | 275/19520 | 0.047960296 | 0.049464532 |
| CC | GO:0044232 | organelle membrane contact site | 39/19520 | 0.048798663 | 0.049464532 |
| MF | GO:0032266 | phosphatidylinositol-3-phosphate binding | 42/18337 | 1.61E-09 | 3.05E-08 |
| MF | GO:1901981 | phosphatidylinositol phosphate binding | 171/18337 | 1.95E-06 | 1.85E-05 |
| MF | GO:0035091 | phosphatidylinositol binding | 253/18337 | 1.32E-05 | 8.34E-05 |
| MF | GO:0051020 | GTPase binding | 222/18337 | 0.00015453 | 0.000731986 |
| MF | GO:0005543 | phospholipid binding | 451/18337 | 0.000205323 | 0.000778067 |
| MF | GO:0080025 | phosphatidylinositol-3,5-bisphosphate binding | 27/18337 | 0.000518247 | 0.001636569 |
| MF | GO:1902936 | phosphatidylinositol bisphosphate binding | 105/18337 | 0.007596929 | 0.020563117 |
| MF | GO:0008641 | ubiquitin-like modifier activating enzyme activity | 10/18337 | 0.01247543 | 0.029547072 |
| MF | GO:0070700 | BMP receptor binding | 13/18337 | 0.016188936 | 0.034081972 |
| MF | GO:0008234 | cysteine-type peptidase activity | 174/18337 | 0.019874611 | 0.037657159 |
| MF | GO:0010314 | phosphatidylinositol-5-phosphate binding | 18/18337 | 0.022348449 | 0.038494935 |
| MF | GO:0070696 | transmembrane receptor protein serine/threonine kinase binding | 24/18337 | 0.029691177 | 0.04294122 |
| MF | GO:0033612 | receptor serine/threonine kinase binding | 28/18337 | 0.034556992 | 0.04294122 |
| MF | GO:0050681 | androgen receptor binding | 28/18337 | 0.034556992 | 0.04294122 |
| MF | GO:0070273 | phosphatidylinositol-4-phosphate binding | 28/18337 | 0.034556992 | 0.04294122 |
| MF | GO:0106311 | protein threonine kinase activity | 248/18337 | 0.038247142 | 0.04294122 |
| MF | GO:0106310 | protein serine kinase activity | 249/18337 | 0.038527817 | 0.04294122 |

**Table 5b. KEGG enrichment analysis.**

| ID | Description | BgRatio | pvalue | qvalue |
| --- | --- | --- | --- | --- |
| hsa04136 | Autophagy - other | 32/8101 | 2.71E-34 | 2.85E-33 |
| hsa04140 | Autophagy - animal | 141/8101 | 1.30E-30 | 6.85E-30 |
| hsa05017 | Spinocerebellar ataxia | 143/8101 | 3.40E-13 | 1.19E-12 |
| hsa05016 | Huntington disease | 306/8101 | 6.75E-10 | 1.78E-09 |
| hsa05014 | Amyotrophic lateral sclerosis | 364/8101 | 3.67E-09 | 7.72E-09 |
| hsa05010 | Alzheimer disease | 384/8101 | 6.16E-09 | 1.08E-08 |
| hsa05022 | Pathways of neurodegeneration - multiple diseases | 476/8101 | 4.84E-08 | 7.28E-08 |
| hsa04137 | Mitophagy - animal | 72/8101 | 2.50E-05 | 3.28E-05 |
| hsa05131 | Shigellosis | 247/8101 | 0.000269466 | 0.000315165 |
| hsa04211 | Longevity regulating pathway | 89/8101 | 0.001276471 | 0.001343654 |

**Appendix 6**

**gene set enrichment analyses (GSEA)**

**Table 6a. GSEA of high rish.**

| NAME | ES | NES | NOM p-val | FDR q-val |
| --- | --- | --- | --- | --- |
| KEGG_FOCAL_ADHESION | 0.69056875 | 2.4489646 | 0 | 0 |
| KEGG_BASAL_CELL_CARCINOMA | 0.6879567 | 2.3809495 | 0 | 0 |
| KEGG_MAPK_SIGNALING_PATHWAY | 0.5621488 | 2.3744888 | 0 | 0 |
| KEGG_PATHWAYS_IN_CANCER | 0.57787985 | 2.3686042 | 0 | 0 |
| KEGG_COMPLEMENT_AND_COAGULATION_CASCADES | 0.7177043 | 2.3570118 | 0 | 0 |
| KEGG_ECM_RECEPTOR_INTERACTION | 0.7978408 | 2.353843 | 0 | 0 |
| KEGG_AXON_GUIDANCE | 0.61885357 | 2.3413005 | 0 | 0 |
| KEGG_DILATED_CARDIOMYOPATHY | 0.68775946 | 2.3330595 | 0 | 0 |
| KEGG_HEDGEHOG_SIGNALING_PATHWAY | 0.66277415 | 2.3217778 | 0 | 1.70E-04 |
| KEGG_MELANOMA | 0.6165267 | 2.3208523 | 0 | 1.53E-04 |
| KEGG_REGULATION_OF_ACTIN_CYTOSKELETON | 0.58657515 | 2.314095 | 0 | 1.39E-04 |
| KEGG_GAP_JUNCTION | 0.5934445 | 2.3002708 | 0 | 1.27E-04 |
| KEGG_CELL_ADHESION_MOLECULES_CAMS | 0.7257324 | 2.2947822 | 0 | 1.17E-04 |
| KEGG_LEUKOCYTE_TRANSENDOTHELIAL_MIGRATION | 0.638092 | 2.2874827 | 0 | 1.09E-04 |
| KEGG_CALCIUM_SIGNALING_PATHWAY | 0.5841124 | 2.2815518 | 0 | 1.02E-04 |
| KEGG_MELANOGENESIS | 0.5872531 | 2.2809613 | 0 | 9.54E-05 |
| KEGG_HYPERTROPHIC_CARDIOMYOPATHY_HCM | 0.65500474 | 2.2718496 | 0 | 1.82E-04 |
| KEGG_VASCULAR_SMOOTH_MUSCLE_CONTRACTION | 0.60492235 | 2.2570276 | 0 | 1.72E-04 |
| KEGG_CYTOKINE_CYTOKINE_RECEPTOR_INTERACTION | 0.6207728 | 2.255368 | 0 | 1.63E-04 |
| KEGG_JAK_STAT_SIGNALING_PATHWAY | 0.57603896 | 2.2473483 | 0 | 1.90E-04 |
| KEGG_WNT_SIGNALING_PATHWAY | 0.5337051 | 2.2469695 | 0 | 1.81E-04 |
| KEGG_ARRHYTHMOGENIC_RIGHT_VENTRICULAR_CARDIOMYOPATHY_ARVC | 0.6635827 | 2.2249298 | 0 | 2.45E-04 |
| KEGG_GLYCOSAMINOGLYCAN_BIOSYNTHESIS_CHONDROITIN_SULFATE | 0.8250325 | 2.1857274 | 0 | 4.75E-04 |
| KEGG_HEMATOPOIETIC_CELL_LINEAGE | 0.71916217 | 2.1805062 | 0.001976285 | 4.56E-04 |
| KEGG_CHEMOKINE_SIGNALING_PATHWAY | 0.6080201 | 2.152601 | 0 | 7.44E-04 |
| KEGG_NEUROACTIVE_LIGAND_RECEPTOR_INTERACTION | 0.58593196 | 2.1276035 | 0 | 0.001085034 |
| KEGG_NOTCH_SIGNALING_PATHWAY | 0.6549746 | 2.122317 | 0 | 0.001215209 |
| KEGG_VIRAL_MYOCARDITIS | 0.65294737 | 2.093776 | 0.003984064 | 0.001754245 |
| KEGG_GLIOMA | 0.56744045 | 2.0876148 | 0 | 0.00184975 |
| KEGG_TOLL_LIKE_RECEPTOR_SIGNALING_PATHWAY | 0.5587609 | 2.0799978 | 0.001996008 | 0.0021117 |
| KEGG_PROSTATE_CANCER | 0.5487938 | 2.0776818 | 0 | 0.002096885 |
| KEGG_TIGHT_JUNCTION | 0.4940532 | 2.0689158 | 0 | 0.00229688 |
| KEGG_SMALL_CELL_LUNG_CANCER | 0.56473035 | 2.0582929 | 0.003968254 | 0.002713095 |
| KEGG_FC_EPSILON_RI_SIGNALING_PATHWAY | 0.5337667 | 2.0526705 | 0.004040404 | 0.002917181 |
| KEGG_TGF_BETA_SIGNALING_PATHWAY | 0.5469859 | 2.0436008 | 0.002004008 | 0.003131777 |
| KEGG_VEGF_SIGNALING_PATHWAY | 0.5303814 | 2.0403905 | 0.00203252 | 0.003238734 |
| KEGG_GLYCOSAMINOGLYCAN_BIOSYNTHESIS_HEPARAN_SULFATE | 0.6208422 | 2.038765 | 0.001972387 | 0.00322807 |
| KEGG_GNRH_SIGNALING_PATHWAY | 0.5020611 | 2.0332692 | 0.002 | 0.003363416 |
| KEGG_AUTOIMMUNE_THYROID_DISEASE | 0.6788425 | 1.9984783 | 0.003913894 | 0.004555695 |
| KEGG_PRION_DISEASES | 0.618012 | 1.9982812 | 0 | 0.004483221 |
| KEGG_ADHERENS_JUNCTION | 0.57346153 | 1.9974627 | 0.003944773 | 0.004424383 |
| KEGG_FC_GAMMA_R_MEDIATED_PHAGOCYTOSIS | 0.55578524 | 1.9957205 | 0.004 | 0.004378377 |
| KEGG_RENAL_CELL_CARCINOMA | 0.55271 | 1.9905957 | 0 | 0.004531993 |
| KEGG_T_CELL_RECEPTOR_SIGNALING_PATHWAY | 0.54982823 | 1.9897068 | 0.007936508 | 0.004557086 |
| KEGG_LONG_TERM_DEPRESSION | 0.5119594 | 1.9759716 | 0.001937985 | 0.005328777 |
| KEGG_LEISHMANIA_INFECTION | 0.6779242 | 1.9713081 | 0.006147541 | 0.005425897 |
| KEGG_PHOSPHATIDYLINOSITOL_SIGNALING_SYSTEM | 0.53380466 | 1.9705611 | 0.00390625 | 0.005383362 |
| KEGG_MTOR_SIGNALING_PATHWAY | 0.49703377 | 1.9570318 | 0 | 0.005929784 |
| KEGG_B_CELL_RECEPTOR_SIGNALING_PATHWAY | 0.5673761 | 1.950198 | 0.010060363 | 0.006356316 |
| KEGG_PANCREATIC_CANCER | 0.54061747 | 1.9497463 | 0.00203666 | 0.006262829 |
| KEGG_ENDOCYTOSIS | 0.47909075 | 1.9442658 | 0 | 0.006440408 |
| KEGG_TYPE_II_DIABETES_MELLITUS | 0.5493957 | 1.9392629 | 0.004219409 | 0.006610119 |
| KEGG_INOSITOL_PHOSPHATE_METABOLISM | 0.5621692 | 1.9384093 | 0.004056795 | 0.006532398 |
| KEGG_ACUTE_MYELOID_LEUKEMIA | 0.5614647 | 1.9272325 | 0.002004008 | 0.007225651 |
| KEGG_NEUROTROPHIN_SIGNALING_PATHWAY | 0.49223414 | 1.9260119 | 0.004056795 | 0.007213341 |
| KEGG_CHRONIC_MYELOID_LEUKEMIA | 0.52507955 | 1.9046643 | 0.008230452 | 0.008764355 |
| KEGG_INTESTINAL_IMMUNE_NETWORK_FOR_IGA_PRODUCTION | 0.6985033 | 1.871252 | 0.020491803 | 0.011640057 |
| KEGG_NATURAL_KILLER_CELL_MEDIATED_CYTOTOXICITY | 0.52340287 | 1.871008 | 0.014373717 | 0.011495588 |
| KEGG_ERBB_SIGNALING_PATHWAY | 0.4780658 | 1.8697712 | 0.008064516 | 0.011433099 |
| KEGG_PRIMARY_IMMUNODEFICIENCY | 0.69730246 | 1.8571508 | 0.03131524 | 0.012513609 |
| KEGG_ASTHMA | 0.7069512 | 1.837828 | 0.019646365 | 0.014689419 |
| KEGG_ABC_TRANSPORTERS | 0.49091128 | 1.8215252 | 0.003766478 | 0.016483285 |
| KEGG_GLYCOSAMINOGLYCAN_BIOSYNTHESIS_KERATAN_SULFATE | 0.7032657 | 1.81644 | 0.016032064 | 0.01703809 |
| KEGG_RIG_I_LIKE_RECEPTOR_SIGNALING_PATHWAY | 0.46957904 | 1.8160923 | 0.001941748 | 0.01678657 |
| KEGG_ALLOGRAFT_REJECTION | 0.7451425 | 1.8091104 | 0.036750484 | 0.017489074 |
| KEGG_DORSO_VENTRAL_AXIS_FORMATION | 0.58373713 | 1.790738 | 0.015625 | 0.019936837 |
| KEGG_PROGESTERONE_MEDIATED_OOCYTE_MATURATION | 0.44341958 | 1.7821937 | 0.009708738 | 0.020894866 |
| KEGG_COLORECTAL_CANCER | 0.47162402 | 1.7706854 | 0.021782178 | 0.0223183 |
| KEGG_TYPE_I_DIABETES_MELLITUS | 0.6663946 | 1.7617283 | 0.047524754 | 0.023557423 |
| KEGG_INSULIN_SIGNALING_PATHWAY | 0.40085083 | 1.7442902 | 0.007874016 | 0.02620201 |
| KEGG_ALDOSTERONE_REGULATED_SODIUM_REABSORPTION | 0.49470434 | 1.709253 | 0.012219959 | 0.03221041 |
| KEGG_EPITHELIAL_CELL_SIGNALING_IN_HELICOBACTER_PYLORI_INFECTION | 0.46588054 | 1.7076621 | 0.015655577 | 0.03212885 |
| KEGG_ADIPOCYTOKINE_SIGNALING_PATHWAY | 0.45408386 | 1.6965119 | 0.012371134 | 0.034230802 |
| KEGG_NOD_LIKE_RECEPTOR_SIGNALING_PATHWAY | 0.48899156 | 1.6940271 | 0.036893204 | 0.034468576 |
| KEGG_TASTE_TRANSDUCTION | 0.5030888 | 1.6909633 | 0.003831418 | 0.034597993 |
| KEGG_NON_SMALL_CELL_LUNG_CANCER | 0.466856 | 1.6880782 | 0.021611001 | 0.034633175 |
| KEGG_GRAFT_VERSUS_HOST_DISEASE | 0.68549323 | 1.6680404 | 0.08316832 | 0.03861399 |
| KEGG_LYSOSOME | 0.4988739 | 1.6671474 | 0.048582997 | 0.038385026 |
| KEGG_ANTIGEN_PROCESSING_AND_PRESENTATION | 0.5350672 | 1.661558 | 0.07355865 | 0.03934379 |
| KEGG_GLYCOSPHINGOLIPID_BIOSYNTHESIS_GANGLIO_SERIES | 0.5867039 | 1.6476068 | 0.034883723 | 0.04222549 |
| KEGG_PATHOGENIC_ESCHERICHIA_COLI_INFECTION | 0.4685625 | 1.6350936 | 0.037037037 | 0.04492767 |
| KEGG_GLYCOSAMINOGLYCAN_DEGRADATION | 0.5663265 | 1.6094797 | 0.052427184 | 0.05147146 |
| KEGG_VASOPRESSIN_REGULATED_WATER_REABSORPTION | 0.44224828 | 1.6087725 | 0.028735632 | 0.05099442 |
| KEGG_BLADDER_CANCER | 0.45038524 | 1.6038738 | 0.025540275 | 0.051873907 |
| KEGG_ENDOMETRIAL_CANCER | 0.44344214 | 1.5862039 | 0.057654075 | 0.056265775 |
| KEGG_GLYCEROPHOSPHOLIPID_METABOLISM | 0.39093107 | 1.5418894 | 0.03206413 | 0.07021958 |
| KEGG_ARACHIDONIC_ACID_METABOLISM | 0.4065364 | 1.5310304 | 0.027777778 | 0.07375608 |
| KEGG_PHENYLALANINE_METABOLISM | 0.5419371 | 1.5276434 | 0.06849315 | 0.07418966 |
| KEGG_SYSTEMIC_LUPUS_ERYTHEMATOSUS | 0.55113125 | 1.5208768 | 0.1932271 | 0.075504355 |
| KEGG_ETHER_LIPID_METABOLISM | 0.43672696 | 1.5178744 | 0.03557312 | 0.07573301 |
| KEGG_APOPTOSIS | 0.39307466 | 1.4777907 | 0.09090909 | 0.09081942 |
| KEGG_RENIN_ANGIOTENSIN_SYSTEM | 0.49519458 | 1.4641951 | 0.092843324 | 0.09535695 |
| KEGG_LONG_TERM_POTENTIATION | 0.36481664 | 1.4517713 | 0.056420233 | 0.09990732 |
| KEGG_CYTOSOLIC_DNA_SENSING_PATHWAY | 0.37797812 | 1.3739221 | 0.09623431 | 0.13919355 |
| KEGG_O_GLYCAN_BIOSYNTHESIS | 0.4590506 | 1.3737655 | 0.154 | 0.13778308 |
| KEGG_GLYCINE_SERINE_AND_THREONINE_METABOLISM | 0.40998182 | 1.3068134 | 0.15289256 | 0.17682827 |
| KEGG_AMYOTROPHIC_LATERAL_SCLEROSIS_ALS | 0.332073 | 1.2891492 | 0.15655577 | 0.18718448 |
| KEGG_SNARE_INTERACTIONS_IN_VESICULAR_TRANSPORT | 0.36563987 | 1.262389 | 0.19688109 | 0.20411381 |
| KEGG_TYROSINE_METABOLISM | 0.3574239 | 1.1884754 | 0.26070037 | 0.2613316 |
| KEGG_VIBRIO_CHOLERAE_INFECTION | 0.32269353 | 1.1608428 | 0.25686276 | 0.2825691 |
| KEGG_GLYCOSPHINGOLIPID_BIOSYNTHESIS_LACTO_AND_NEOLACTO_SERIES | 0.3488287 | 1.1442919 | 0.30604288 | 0.29469562 |
| KEGG_GLYCEROLIPID_METABOLISM | 0.295822 | 1.1146739 | 0.28629032 | 0.31998736 |
| KEGG_GALACTOSE_METABOLISM | 0.34791085 | 1.1017647 | 0.35177866 | 0.32902357 |
| KEGG_SPHINGOLIPID_METABOLISM | 0.3148997 | 1.0818797 | 0.34552845 | 0.34637058 |
| KEGG_PANTOTHENATE_AND_COA_BIOSYNTHESIS | 0.3455025 | 1.0496795 | 0.3972056 | 0.3768522 |
| KEGG_BETA_ALANINE_METABOLISM | 0.34544387 | 1.045007 | 0.4004193 | 0.37849313 |
| KEGG_PPAR_SIGNALING_PATHWAY | 0.26685905 | 0.98774874 | 0.47379032 | 0.43955514 |
| KEGG_REGULATION_OF_AUTOPHAGY | 0.2629954 | 0.9182546 | 0.6123595 | 0.5222584 |
| KEGG_STARCH_AND_SUCROSE_METABOLISM | 0.24587888 | 0.8757964 | 0.6294821 | 0.57230204 |
| KEGG_STEROID_HORMONE_BIOSYNTHESIS | 0.23077184 | 0.8080532 | 0.731569 | 0.6602683 |
| KEGG_OLFACTORY_TRANSDUCTION | 0.2536078 | 0.7149428 | 0.7923387 | 0.7793302 |

**Table 6b. GSEA of low rish.**

| NAME | ES | NES | NOM p-val | FDR q-val |
| --- | --- | --- | --- | --- |
| KEGG_PEROXISOME | -0.6998327 | -2.3484135 | 0 | 0 |
| KEGG_PYRUVATE_METABOLISM | -0.6739246 | -2.0967762 | 0 | 0.009004418 |
| KEGG_OXIDATIVE_PHOSPHORYLATION | -0.72717154 | -2.047305 | 0 | 0.011778654 |
| KEGG_PARKINSONS_DISEASE | -0.7032633 | -2.000377 | 0 | 0.01592972 |
| KEGG_PROPANOATE_METABOLISM | -0.69930387 | -1.99876 | 0.00189394 | 0.013292935 |
| KEGG_HUNTINGTONS_DISEASE | -0.582352 | -1.9861877 | 0 | 0.013819993 |
| KEGG_ALZHEIMERS_DISEASE | -0.5721536 | -1.9739902 | 0 | 0.013674793 |
| KEGG_CITRATE_CYCLE_TCA_CYCLE | -0.7870222 | -1.9547057 | 0 | 0.014503794 |
| KEGG_AMINOACYL_TRNA_BIOSYNTHESIS | -0.72552097 | -1.9062151 | 0.009191177 | 0.024714792 |
| KEGG_RIBOSOME | -0.8629255 | -1.8607721 | 0.00204499 | 0.033994645 |
| KEGG_TERPENOID_BACKBONE_BIOSYNTHESIS | -0.7987688 | -1.8531097 | 0.007889546 | 0.03301008 |
| KEGG_FATTY_ACID_METABOLISM | -0.5869374 | -1.8469139 | 0.005825243 | 0.03305446 |
| KEGG_PROTEASOME | -0.72097754 | -1.8273118 | 0.009765625 | 0.036341853 |
| KEGG_MISMATCH_REPAIR | -0.7033247 | -1.8259623 | 0.019569471 | 0.034121532 |
| KEGG_BUTANOATE_METABOLISM | -0.6272313 | -1.7873236 | 0.015594542 | 0.04585408 |
| KEGG_DNA_REPLICATION | -0.72673136 | -1.774758 | 0.023762377 | 0.04821093 |
| KEGG_VALINE_LEUCINE_AND_ISOLEUCINE_DEGRADATION | -0.6562798 | -1.7714092 | 0.023391813 | 0.046338078 |
| KEGG_RNA_DEGRADATION | -0.5306977 | -1.7582322 | 0.027237354 | 0.048726805 |
| KEGG_GLYOXYLATE_AND_DICARBOXYLATE_METABOLISM | -0.66110724 | -1.7449366 | 0.022044089 | 0.051344503 |
| KEGG_PENTOSE_PHOSPHATE_PATHWAY | -0.60522777 | -1.731082 | 0.026 | 0.054254573 |
| KEGG_BASE_EXCISION_REPAIR | -0.6115172 | -1.6779987 | 0.039215688 | 0.07628757 |
| KEGG_NUCLEOTIDE_EXCISION_REPAIR | -0.5655234 | -1.6735423 | 0.0625 | 0.07456996 |
| KEGG_GLYCOSYLPHOSPHATIDYLINOSITOL_GPI_ANCHOR_BIOSYNTHESIS | -0.5608249 | -1.665201 | 0.056485355 | 0.07575006 |
| KEGG_STEROID_BIOSYNTHESIS | -0.69074595 | -1.6475202 | 0.03941909 | 0.08126035 |
| KEGG_N_GLYCAN_BIOSYNTHESIS | -0.4781336 | -1.6416683 | 0.03960396 | 0.08050994 |
| KEGG_GLUTATHIONE_METABOLISM | -0.5044582 | -1.6228296 | 0.036885247 | 0.08705415 |
| KEGG_SELENOAMINO_ACID_METABOLISM | -0.5399932 | -1.5877584 | 0.06358381 | 0.10227417 |
| KEGG_PYRIMIDINE_METABOLISM | -0.4635017 | -1.5851077 | 0.042424243 | 0.10023604 |
| KEGG_CELL_CYCLE | -0.46832564 | -1.583091 | 0.08565737 | 0.09787485 |
| KEGG_ALANINE_ASPARTATE_AND_GLUTAMATE_METABOLISM | -0.4534747 | -1.5012821 | 0.046 | 0.14339985 |
| KEGG_BIOSYNTHESIS_OF_UNSATURATED_FATTY_ACIDS | -0.50361294 | -1.498771 | 0.07254902 | 0.14059252 |
| KEGG_CYSTEINE_AND_METHIONINE_METABOLISM | -0.42260224 | -1.4577473 | 0.07633588 | 0.1648239 |
| KEGG_SPLICEOSOME | -0.49006438 | -1.4506661 | 0.14587332 | 0.16567963 |
| KEGG_PROTEIN_EXPORT | -0.5601813 | -1.4272133 | 0.16232465 | 0.1793068 |
| KEGG_GLYCOLYSIS_GLUCONEOGENESIS | -0.43299487 | -1.407572 | 0.12720157 | 0.1904751 |
| KEGG_OOCYTE_MEIOSIS | -0.36144254 | -1.3986443 | 0.13855422 | 0.1922432 |
| KEGG_UBIQUITIN_MEDIATED_PROTEOLYSIS | -0.3477171 | -1.3726007 | 0.1529175 | 0.20908539 |
| KEGG_PROXIMAL_TUBULE_BICARBONATE_RECLAMATION | -0.43571255 | -1.369507 | 0.096525095 | 0.20648283 |
| KEGG_BASAL_TRANSCRIPTION_FACTORS | -0.44664866 | -1.3691462 | 0.16734694 | 0.20149894 |
| KEGG_PURINE_METABOLISM | -0.3152773 | -1.3290707 | 0.12825651 | 0.2322141 |
| KEGG_ARGININE_AND_PROLINE_METABOLISM | -0.39846477 | -1.3270578 | 0.13584906 | 0.22854547 |
| KEGG_AMINO_SUGAR_AND_NUCLEOTIDE_SUGAR_METABOLISM | -0.39926526 | -1.30745 | 0.1602434 | 0.24075341 |
| KEGG_ONE_CARBON_POOL_BY_FOLATE | -0.5091049 | -1.2754844 | 0.22200392 | 0.26590967 |
| KEGG_RNA_POLYMERASE | -0.45997575 | -1.263642 | 0.23062016 | 0.2714819 |
| KEGG_CARDIAC_MUSCLE_CONTRACTION | -0.36971247 | -1.2612947 | 0.18164062 | 0.26783 |
| KEGG_DRUG_METABOLISM_OTHER_ENZYMES | -0.34153673 | -1.2530609 | 0.1733871 | 0.2694906 |
| KEGG_PRIMARY_BILE_ACID_BIOSYNTHESIS | -0.40711847 | -1.218853 | 0.2188755 | 0.29856676 |
| KEGG_FRUCTOSE_AND_MANNOSE_METABOLISM | -0.38722473 | -1.1904001 | 0.2871486 | 0.32244405 |
| KEGG_OTHER_GLYCAN_DEGRADATION | -0.45085657 | -1.1822586 | 0.27111983 | 0.32366282 |
| KEGG_PENTOSE_AND_GLUCURONATE_INTERCONVERSIONS | -0.39147115 | -1.1707859 | 0.25779626 | 0.3288856 |
| KEGG_P53_SIGNALING_PATHWAY | -0.31373298 | -1.1653719 | 0.25306123 | 0.32804853 |
| KEGG_PORPHYRIN_AND_CHLOROPHYLL_METABOLISM | -0.3569011 | -1.1624556 | 0.26104417 | 0.3247215 |
| KEGG_THYROID_CANCER | -0.3360959 | -1.1356802 | 0.30177516 | 0.34806803 |
| KEGG_NICOTINATE_AND_NICOTINAMIDE_METABOLISM | -0.3283276 | -1.1325622 | 0.28456914 | 0.34466022 |
| KEGG_TRYPTOPHAN_METABOLISM | -0.33878297 | -1.1318468 | 0.26796117 | 0.33914766 |
| KEGG_RIBOFLAVIN_METABOLISM | -0.34331372 | -1.0919691 | 0.3449692 | 0.37835127 |
| KEGG_HISTIDINE_METABOLISM | -0.34508073 | -1.0534275 | 0.38446215 | 0.41630393 |
| KEGG_LYSINE_DEGRADATION | -0.31121278 | -1.0285219 | 0.41793892 | 0.43945336 |
| KEGG_HOMOLOGOUS_RECOMBINATION | -0.35419723 | -1.0007236 | 0.44580778 | 0.46729246 |
| KEGG_METABOLISM_OF_XENOBIOTICS_BY_CYTOCHROME_P450 | -0.2815621 | -0.9957268 | 0.44008264 | 0.46621552 |
| KEGG_ASCORBATE_AND_ALDARATE_METABOLISM | -0.3495784 | -0.987174 | 0.469428 | 0.46924654 |
| KEGG_ALPHA_LINOLENIC_ACID_METABOLISM | -0.3314563 | -0.96998274 | 0.5066922 | 0.48337933 |
| KEGG_MATURITY_ONSET_DIABETES_OF_THE_YOUNG | -0.33698285 | -0.9595183 | 0.4939759 | 0.48936942 |
| KEGG_LINOLEIC_ACID_METABOLISM | -0.28325945 | -0.9165051 | 0.56143665 | 0.5373829 |
| KEGG_DRUG_METABOLISM_CYTOCHROME_P450 | -0.24461308 | -0.87547266 | 0.60248446 | 0.5827337 |
| KEGG_RETINOL_METABOLISM | -0.23413691 | -0.82065976 | 0.7016129 | 0.64899725 |
| KEGG_NITROGEN_METABOLISM | -0.24718589 | -0.7922155 | 0.766129 | 0.67733324 |
